# Autonomic dysfunction and hemodynamic management after acute spinal cord injury: blood pressure targets, perfusion strategies, and emerging therapies

**DOI:** 10.3389/fneur.2025.1716013

**Published:** 2026-01-06

**Authors:** David Travis Johnston, James W. Grau

**Affiliations:** 1 McGovern Medical School at UTHealth Houston, Houston, TX, United States; 2 Texas A&M University, College Station, TX, United States

**Keywords:** autonomic dysfunction, hemodynamic management, mean arterial pressure, neurocritical care, neuromodulation, spinal cord injury, spinal cord perfusion pressure, vasopressors

## Abstract

**Objective:**

Acute spinal cord injury (SCI) produces profound cardiovascular instability that exacerbates secondary damage, emphasizing the need for timely blood pressure management and hemodynamic support. While stabilizing hemodynamics is central to acute SCI management, evidence guiding optimal mean arterial pressure (MAP) targets, vasopressor selection, and management strategies remains limited. We conducted a narrative, comprehensive review of peer-reviewed clinical and preclinical studies addressing hemodynamic management after SCI, defined here as the first 7 days after injury, including MAP augmentation, spinal cord perfusion pressure (SCPP) monitoring, vasopressor selection, and neuromodulatory approaches.

**Results:**

Observational studies show that even transient hypotensive episodes within the first 72 h worsen neurological recovery. Updated guidelines recommend maintaining MAP between 75 to 80 and 90 to 95 mmHg for 3 to 7 days following injury. Norepinephrine is favored as first-line therapy because it reliably raises MAP with fewer adverse effects than other vasopressors. Neuromodulation with tSCS or eSCS has been shown to restore blood pressure and stabilize cardiovascular control in chronic SCI. Emerging evidence suggest these neuromodulatory approaches may be adapted for acute care. SCPP-guided strategies using lumbar cerebrospinal fluid drainage or direct intraspinal monitoring better reflect local perfusion and predict outcomes more accurately than MAP alone, although their use is limited to specialized centers.

**Conclusion:**

Hemodynamic management after SCI should be considered a therapeutic intervention that directly modifies secondary injury mechanisms. Refining MAP targets, expanding access to SCPP-guided care, and evaluating staged neuromodulation, could enhance precision and individualized care to improve long-term recovery. Large-scale multicenter trials will be essential to establish protocols that improve both neurological and cardiovascular outcomes after SCI.

## 1 Introduction

Spinal cord injury (SCI) affects more than 300,000 people in the United States, with an average of 18,000 new cases each year (1, 2). These injuries produce lasting sensory, motor, and autonomic sequelae that diminish independence and quality of life (3, 4). Sensory and motor impairments generally reflect the neurological level and completeness of injury, which provides clinicians with a practical framework for treatment and prognosis (3). In contrast, autonomic dysfunction, particularly cardiovascular dysregulation, demonstrates weak and inconsistent association with neurological level and frequently occurs independently of motor and sensory deficit severity (5–7). Recent work demonstrates that the spinal cord region affected (neurological level) and American Spinal Injury Association (ASIA) Impairment Scale do not reliably predict cardiovascular instability after SCI, underscoring the need for direct autonomic assessment to better identify patients at risk of secondary complications (5). This divergence emphasizes that autonomic impairment follows a distinct pathophysiology from sensory and motor loss and highlights the need for targeted approaches to hemodynamic management after SCI.

In the immediate aftermath of an acute SCI, disruption of sympathetic autonomic pathways often leads to severe hemodynamic instability. Higher level injuries can produce unopposed parasympathetic vagal activity, resulting in hypotension and bradycardia, characteristics of neurogenic shock (8). Further, the lack of blood pressure autoregulation in the hours to days after injury increases the risk of secondary ischemic damage, worsens neurologic outcomes, and contributes to preventable mortality (9–11). Hypotension and hypoxia are well known secondary insults that worsen the extent of spinal cord damage, making aggressive hemodynamic management a mainstay of acute SCI care (12, 13).

Augmenting systemic blood pressure during the acute phase of SCI improves spinal cord blood flow to minimize further ischemic insult to the cord and is associated with improved neurological recovery (11, 14, 15). The earliest evidence that systemic blood pressure influences spinal cord blood flow dates back to the 1970’s (16), with the first studies targeting blood pressure to induce neuroprotection occurring soon after (17). Despite decades of research, high-quality studies to define blood pressure targets, monitoring techniques for perfusion, and vasopressor choice, remain limited. This in turn has weakened guideline recommendations and led to wide variability in clinical implementation (18–22).

For these reasons, there is a pressing need to clarify best practices for hemodynamic management in acute SCI. This review is structured as a narrative synthesis of peer-reviewed clinical and pre-clinical evidence to address those gaps in knowledge. Throughout this review, we use “acute” to denote the first 7 days after injury. This operational window reflects the period in which secondary ischemic and inflammatory mechanisms are most active and is consistent with prior pathophysiologic and guideline frameworks that recommend augmenting blood pressure for roughly the first week after injury (10, 22–25). We first provide a brief overview of normal autonomic control of cardiovascular function and describe how acute SCI disrupts this regulation and affects secondary injury processes to undermine neurological recovery. We then discuss current management strategies including targeting mean arterial pressure (MAP), vasopressor selection, and recent work supporting guideline updates. Next, emerging techniques that aim to improve spinal cord perfusion through pharmacologic and device-based interventions are reviewed for their translational potential. Finally, we highlight key unresolved questions in the field including the uncertainty in optimal blood pressure targets and the development of improved monitoring and techniques. This review aims to highlight the ongoing and completed work that is guiding the constantly evolving area of hemodynamic management in acute SCI. Further, we hope to bolster understanding in this area to motivate future research and guide clinicians toward better acute SCI care strategies. Recent AO Spine Knowledge Forum reports extend this framework to surgical decision making in acute traumatic SCI, emphasizing early decompression when feasible and contemporary operative strategies that are implemented within guideline-informed hemodynamic care (25, 26).

Most of the clinical and preclinical evidence that underpins current hemodynamic management guidelines comes from cohorts with acute traumatic SCI. However, similar patterns of autonomic dysfunction, blood pressure lability, and perfusion-related secondary injury are reported across traumatic and non-traumatic SCI, reflecting shared disruption of supraspinal sympathetic control (3, 27, 28). Noniatrogenic vascular causes of spinal cord ischemia, including infarction, are frequently associated with aortic pathology and are typically managed with blood pressure augmentation and in select cases, cerebrospinal fluid (CSF) drainage to support spinal cord perfusion (29). Together, these shared pathophysiologic features suggest that principles of blood pressure optimization and perfusion targeted management are mechanistically relevant across acute SCI presentations, even though guideline development has largely been driven by traumatic cohorts and dedicated validation in non-traumatic injury remains limited (22, 29).

## 2 Autonomic control of hemodynamics

**Figure 1:**
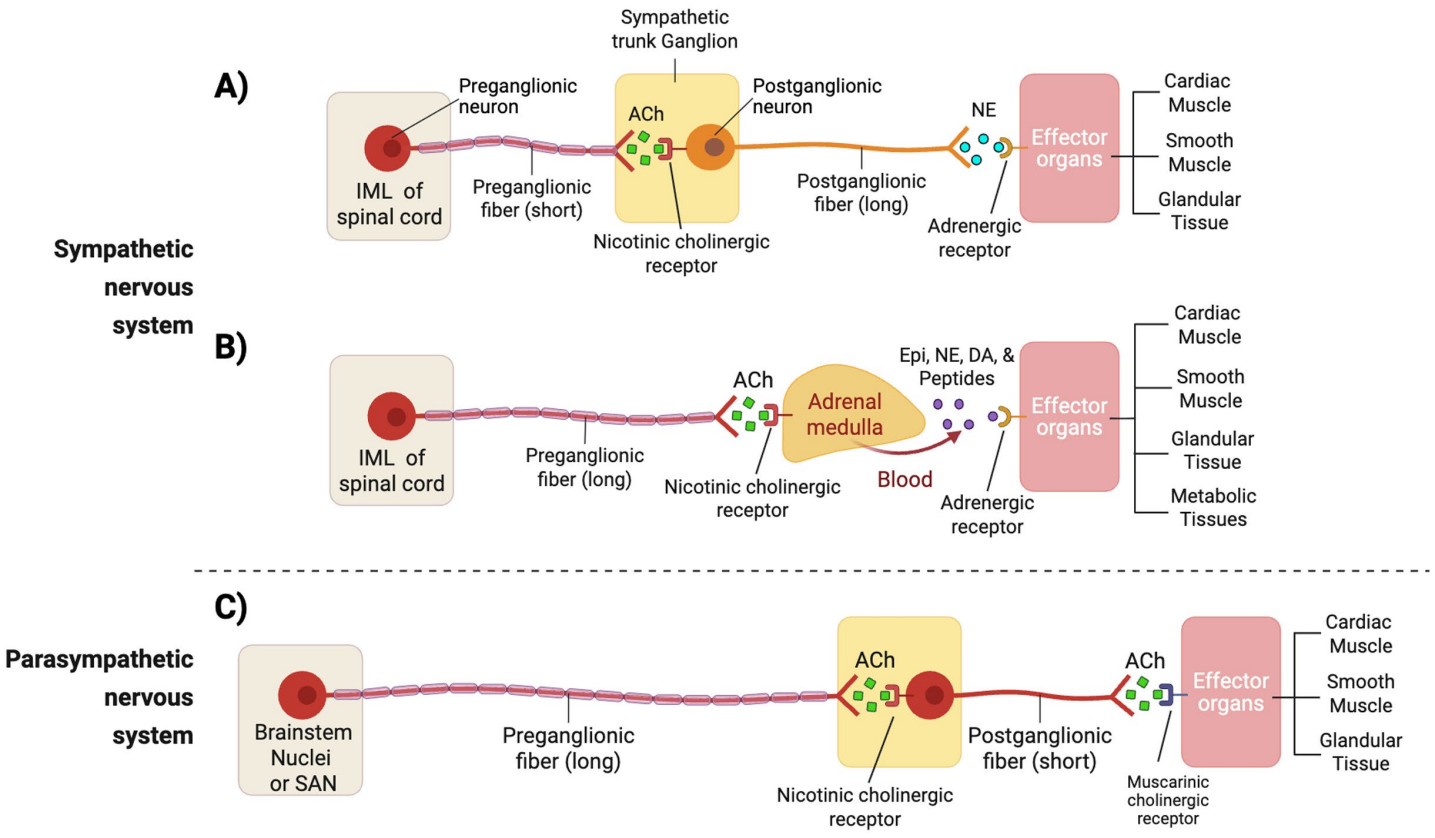
Autonomic nervous system innervation. The autonomic nervous system consists of the sympathetic and parasympathetic divisions: **(A)** Sympathetic pathway via chain ganglia. Preganglionic neurons originate in intermediolateral cell column (IML) of the spinal cord gray matter (T1-L2). Their axons exit via ventral roots, pass into spinal nerves, and enter the sympathetic chain through the white rami communicantes (myelinated preganglionic fibers). Here, preganglionic axons synapse on postganglionic neurons, releasing acetylcholine (ACh) onto nicotinic receptors. Postganglionic axons then exit the sympathetic chain, rejoin spinal nerves through the gray rami communicantes (unmyelinated fibers present at all levels), and project to effector organs, where they primarily release norepinephrine (NE) onto adrenergic receptors. **(B)** Sympathetic pathway via adrenal medulla. Preganglionic axons from the IML project directly to the adrenal medulla, releasing ACh onto nicotinic receptors of chromaffin cells, which then secrete epinephrine (and norepinephrine, dopamine, peptides) into the blood stream to exert a broad systemic effect by acting on adrenergic receptors of various effector organs. **(C)** Parasympathetic pathway. Parasympathetic preganglionic neurons originate from either brainstem nuclei (Cranial nerves III, VII, IX, and X) or from the spinal cord sacral autonomic nucleus (SAN; S2-S4). Their long preganglionic axons synapse in ganglia located near or within target organs, releasing ACh onto nicotinic receptors. Short postganglionic fibers then release acetylcholine (ACh) onto muscarinic receptors at effector organs. Source: Gordan 2015 (30); Loewy 1990 (42); Purves 2018 (32); Westfall 2011 (41). Created with BioRender.com. ACh, acetylcholine; IML, intermediolateral cell column; SAN, sacral autonomic nucleus; NE, norepinephrine.

**Figure 2:**
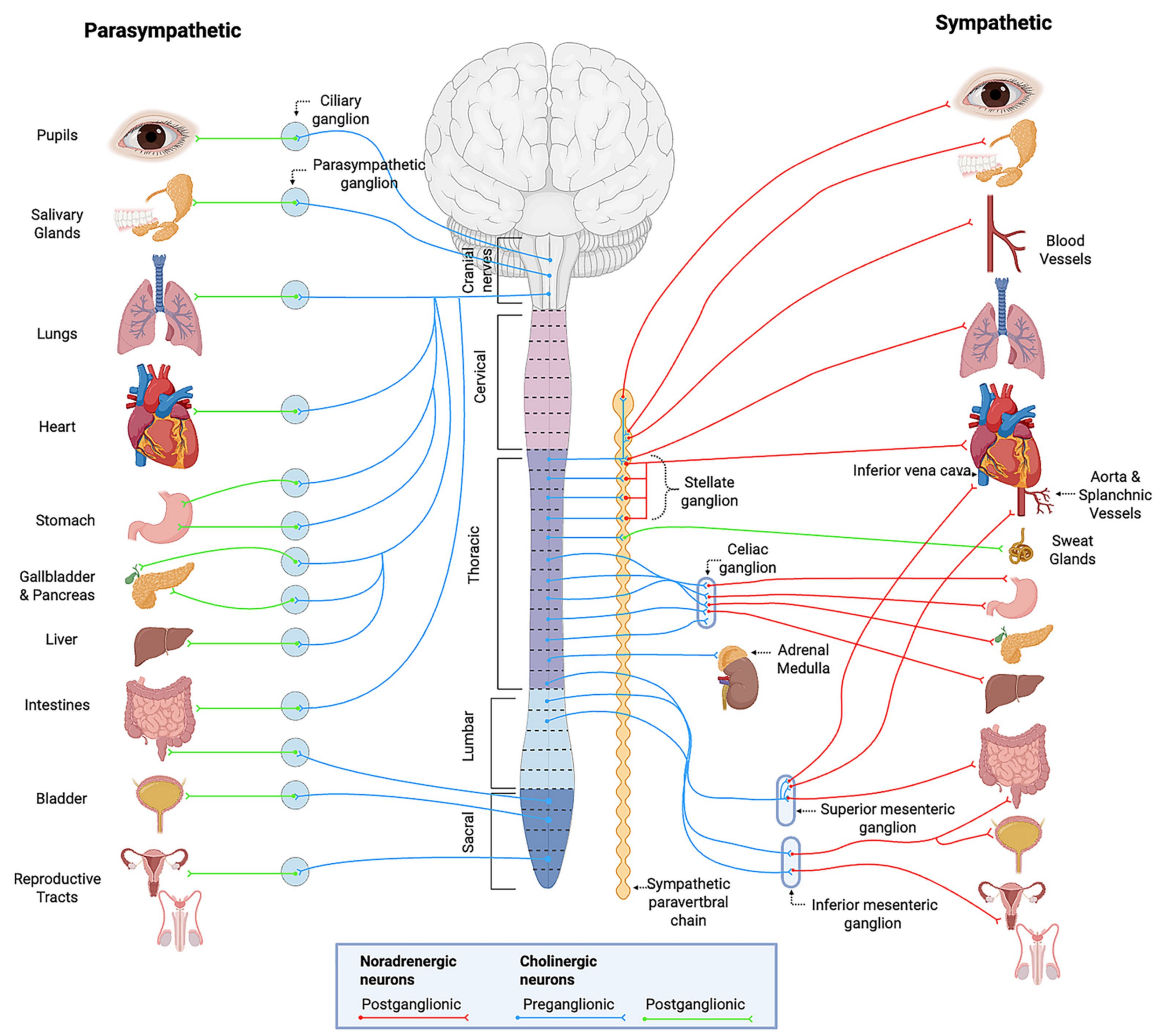
Overview of the autonomic nervous system. General schematic of craniosacral parasympathetic and thoracolumbar sympathetic divisions, showing preganglionic and postganglionic pathways, associated ganglia, and effector organ targets. Provides context for organ-level func7ons summarized in Table 2. Created with BioRender.com. Adapted from Purves 2018 (32).

The autonomic nervous system maintains cardiovascular homeostasis by regulating heart rate, vascular tone, and cardiac output through both rapid reflex arcs and longer-term neuroendocrine mechanisms (30–32). It consists of two primary branches: the sympathetic and parasympathetic nervous systems, which exert opposing effects on cardiovascular function (Figure 1). The sympathetic nervous system increases heart rate, myocardial contractility, and vasoconstriction via the release of norepinephrine (NE) and epinephrine, primarily through β_1_ and α_1_ adrenergic receptors (33–35) (Table 1). In contrast, the parasympathetic nervous system slows the heart rate and reduces atrioventricular nodal conduction mediated primarily by the vagus nerve and subsequent release of acetylcholine (ACh), which engages M_2_ muscarinic receptors (36). This coordinated balance between sympathetic and parasympathetic activity also influences numerous organ systems illustrated in Figure 2 and summarized in Table 2. The remainder of this section will focus on the autonomic control of hemodynamic function with integral neural structures and pathways discussed below.

**Table 1:** Autonomic and neuroendocrine receptors relevant to hemodynamic control.

Autonomic branch	Receptor	Endogenous agonist(s)	Primary sites (hemodynamic specific)	Primary hemodynamic effect
Sympathetic	α1-adrenergic	Norepinephrine, epinephrine	Arterioles/venous capacitance vessels; skin, splanchnic, renal; some coronary	Vasoconstriction; ↑ systemic vascular resistance; ↑ venous return
Sympathetic	α2-adrenergic	Norepinephrine, epinephrine	Presynaptic terminals; vascular smooth muscle	↓ NE release (presynaptic); modest vasoconstriction postsynaptically
Sympathetic	β1-adrenergic	Norepinephrine > epinephrine	SA/AV nodes, atria, ventricles; juxtaglomerular cells	↑ Heart rate, ↑ conduction, ↑ contractility; ↑ renin release
Sympathetic	β2-adrenergic	Epinephrine ≥ norepinephrine	Skeletal muscle arterioles, coronary and hepatic vasculature; bronchi	Vasodilation in select beds; bronchodilation
Sympathetic	D1 dopaminergic	Dopamine	Renal, mesenteric, coronary, cerebral arterioles	Vasodilation and ↑ renal blood flow at low doses
Parasympathetic	M2 muscarinic	Acetylcholine	SA/AV nodes; atrial myocardium	↓ Heart rate and AV nodal conduction
Parasympathetic	M3 muscarinic	Acetylcholine	Endothelial cells of systemic/coronary vessels; vascular smooth muscle	Endothelium-dependent vasodilation via NO; context-dependent vasoconstriction if endothelium injured
Neuroendocrine	Angiotensin II (AT1 receptor)	Angiotensin II	Vascular smooth muscle; adrenal cortex; brainstem/hypothalamus	Vasoconstriction; ↑ aldosterone; ↑ central sympathetic outflow; ↓ baroreflex sensitivity
Neuroendocrine	Angiotensin II (AT2 receptor)	Angiotensin II	Endothelium; adrenal medulla; CNS (developmental prominence)	Counter-regulatory vasodilation, natriuresis, and anti-proliferative effects
Neuroendocrine	Arginine vasopressin (V1A)	Arginine vasopressin	Vascular smooth muscle (systemic, splanchnic)	Vasoconstriction; ↑ MAP
Neuroendocrine	Arginine vasopressin (V2)	Arginine vasopressin	Renal collecting duct	↑ Water reabsorption; ↑ intravascular volume

A summary of autonomic receptor subtypes, endogenous agonists, primary effector sites, and hemodynamic effects. Sources: Brunton 2018 (34); Hall 2021 (35); Westfall 2011 (41). MAP, mean arterial pressure; SA, sinoatrial; AV, atrioventricular; NO, nitric oxide.

**Table 2:** Functions of the autonomic nervous system.

Effector organ	Parasympathetic action	Sympathetic action and adrenergic receptor type
Heart	Decreases heart rate; decreases contractility (atria)	Increases heart rate and contractility (β1)
Arterioles	No significant effect	Constriction (α1, α2); dilation (β2)
Veins	No significant effect	Constriction (α1, α2); dilation (β2)
Lungs (bronchioles, glands)	Constriction; stimulates secretion	Relaxation (β2); inhibits secretion (α1)
Gastrointestinal tract	Increases motility and secretion	Decreases motility and secretion (α1, α2, β2); constricts sphincters (α1)
Liver	No significant effect	Glycogenolysis and gluconeogenesis (α1, β2)
Pancreas	Stimulates insulin secretion	Inhibits insulin secretion (α2)
Adrenal medulla	No significant effect	Secretes epinephrine and norepinephrine (nicotinic receptors)
Kidney	No significant effect	Secretes renin (β1)
Bladder	Contracts detrusor; relaxes sphincter	Relaxes detrusor (β2); contracts sphincter (α1)
Male genitalia	Erection (via Nitric Oxide)	Ejaculation (α1)
Female genitalia (uterus)	Variable (depends on cycle stage)	Contraction (α1); relaxation (β2)
Sweat glands	No significant effect	Generalized secretion (muscarinic); localized secretion (α1)
Salivary glands	Stimulates secretion (watery)	Stimulates secretion (viscous, α1)
Eye (iris, pupil)	Contracts circular muscle → miosis	Contracts radial muscle (α1) → mydriasis
Ciliary muscle (lens)	Contracts for near vision	Relaxes for far vision (β2)
Skin (piloerector muscles)	No significant effect	Contraction (α1)

General summary of parasympathetic and sympathetic actions across effector organs. Sources: Gordan 2015 (30); Loewy 1990 (42); Purves 2018 (32); Westfall 2011 (41).

### 2.1 Baroreceptor reflex

**Figure 3:**
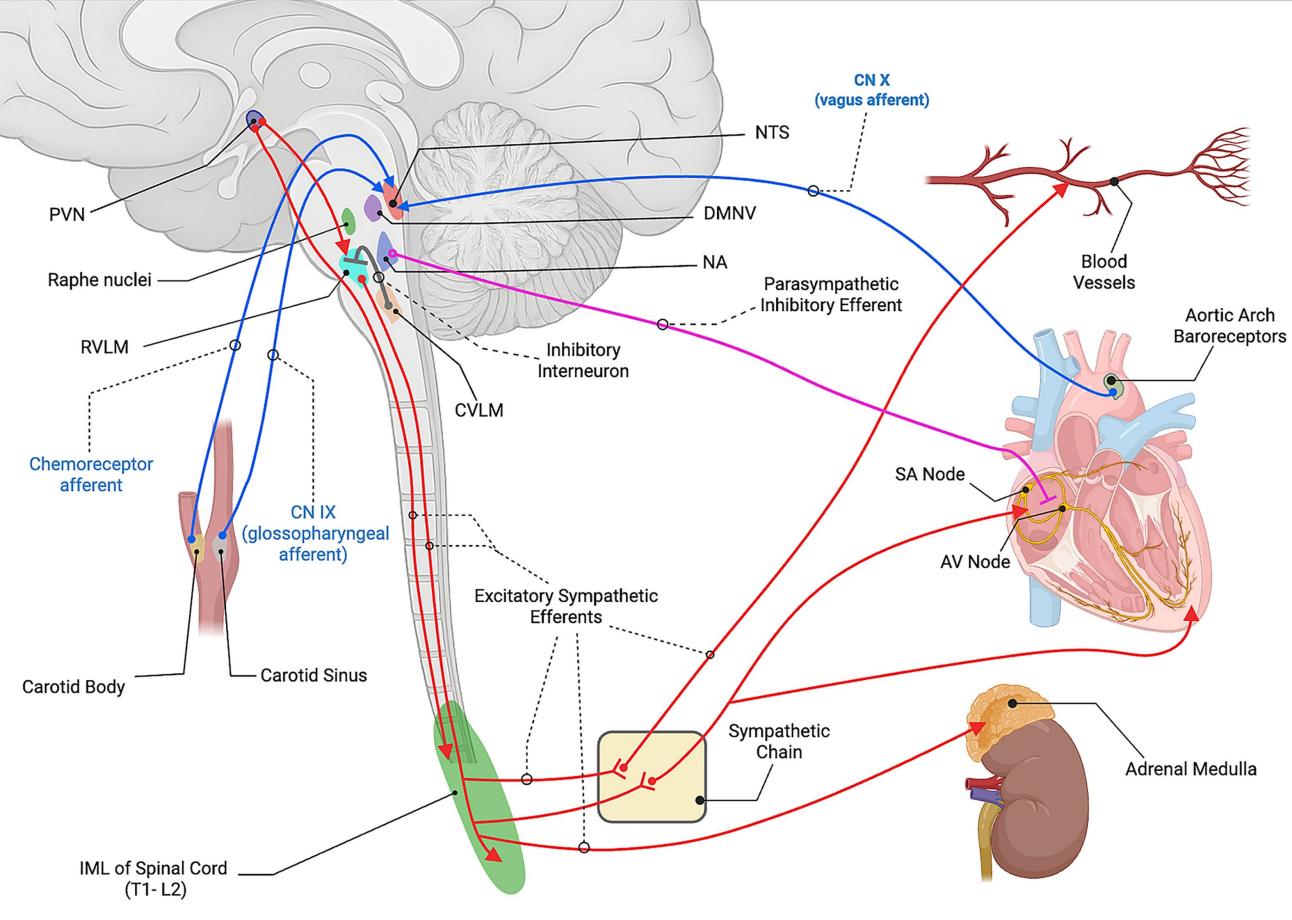
Neural control of cardiovascular function. Baroreceptor afferents from the carotid sinus (CN IX) and aortic arch (CN X), and chemoreceptor afferents from the carotid body, project to the nucleus tractus solitarius (NTS). The NTS activates parasympathetic cardioinhibitory neurons in the nucleus ambiguus (NA) and dorsal motor nucleus of the vagus (DMNV). In parallel, the NTS activates the caudal ventrolateral medulla (CVLM), which inhibits the rostral ventrolateral medulla (RVLM) through interneuron projections. The paraventricular nucleus (PVN) of the hypothalamus provides descending excitatory input to the RVLM as well as directly to the intermediolateral cell column (IML) of the spinal cord (T1-L2 overall, with cardiac sympathetic outflow primarily from T1-T5). RVLM premotor neurons drive sympathetic outflow through the IML and synapse with postganglionic fibers at the sympathetic chain that then travel to the heart and blood vessels. These postganglionic fibers release norepinephrine (NE) onto β1- adrenergic receptors in the sinoatrial node, atrioventricular node, ventricular myocardium increasing heart rate, conduction velocity, and contractility. NE also acts on the α1-adrenergic receptors on vascular smooth muscle to produce vasoconstriction. Furthermore, a subset of preganglionic fibers project directly from the IML to the adrenal medulla, where acetylcholine (ACh) activates nicotinic receptors on chromaffin cells, stimulating the release of epinephrine (Epi) and NE into the circulation. Parasympathetic vagal efferents from the NA release ACh onto muscarinic (M2) receptors in the heart to slow rate and conduction. Created with BioRender.com. Source: Dampney 1994 (37); Guyenet 2006 (38); Guyenet and Stornetta 2022 (39); Loewy 1990 (42). CN, cranial nerve; NTS, nucleus tractus solitarius; DMNV, dorsal motor nucleus of the vagus; NA, nucleus ambiguus; CVLM, caudal ventrolateral medulla; RVLM, rostral ventrolateral medulla; PVN, paraventricular nucleus; IML, intermediolateral cell column; SA node, sinoatrial node; AV node, atrioventricular node; NE, norepinephrine; ACh, acetylcholine; Epi, epinephrine.

The baroreceptor reflex is the principal mechanism for maintaining arterial pressure in response to rapid pressure changes (Figure 3). High-pressure baroreceptors located in the carotid sinus and aortic arch detect changes in arterial stretch and signal alterations via cranial nerves IX and X, respectively, to the nucleus tractus solitarius (NTS) in the dorsal medulla (37). The NTS serves as the primary integration area and relays excitatory glutamatergic input to two downstream centers. The first activates cardioinhibitory parasympathetic neurons in the nucleus ambiguus (NA) and dorsal motor nucleus of the vagus (DMNV), which release ACh to slow the heart via M_2_ receptors (30). The second activates the caudal ventrolateral medulla (CVLM), which contains GABAergic neurons that inhibit the rostral ventrolateral medulla (RVLM), the primary excitatory output center for the sympathetic nervous system (38–40).

When activated, signals from the RVLM descend ipsilaterally through the reticulospinal tract and terminate in the intermediolateral cell column within the T1-L2 lateral horn of the spinal cord gray matter. These neurons release glutamate to activate sympathetic preganglionic neurons, which in turn engage postganglionic neurons that release NE at peripheral sites. Furthermore, these postganglionic fibers stimulate the adrenal medulla to release epinephrine, enhancing vasoconstriction and cardiac output (33, 41).

### 2.2 Parasympathetic activity

Parasympathetic control of the heart is mediated through vagal efferents originating in the NA and DMNV. These neurons synapse on cardiac ganglia and release ACh, which binds to M_2_ receptors to activate G-protein-coupled inward rectifier potassium channels, which slows heart rate and conduction (30). While the NA is primarily involved in acute parasympathetic regulation, the DMNV contributes more broadly to visceral autonomic output (31, 36).

### 2.3 Supraspinal circuitry

Supraspinal structures such as the hypothalamus, periaqueductal gray (PAG), and limbic regions further refine autonomic control by integrating emotional, thermoregulatory, and endocrine signals (42).

The paraventricular nucleus (PVN) of the hypothalamus sends glutamatergic and vasopressinergic projections to the RVLM and spinal cord to increase sympathetic drive, especially during dehydration, stress, or hypovolemia (33, 43). The PVN also modulates baroreflex sensitivity and releases arginine vasopressin, which acts on vascular V1 receptors to promote vasoconstriction and renal V2 receptors to enhance water reabsorption (44).

The PAG is a midbrain structure that contributes to both autonomic responses and descending analgesia, using serotonin (5-HT) projections from the raphe nuclei to modulate tonic sympathetic tone across circadian cycles (45, 46). Its dorsolateral columns initiate sympathetic activation during active coping (fight-or-flight), while ventrolateral regions promote parasympathetic responses (bradycardia, hypotension) associated with passive coping strategies (47).

Limbic structures, including the amygdala, anterior cingulate cortex, and insular cortex process affective and visceral sensory inputs (48). These structures modulate downstream autonomic output through projections to the hypothalamus and PAG.

### 2.4 Renin-angiotensin-aldosterone system

The renin-angiotensin-aldosterone endocrine system responds to a loss in blood pressure through the expression of angiotensin II, which augments central sympathetic outflow by acting on hypothalamic and brainstem centers and dampening baroreflex sensitivity, leading to elevated sympathetic tone (33, 45). In parallel, aldosterone promotes sodium reabsorption in the renal distal tubules, leading to intravascular volume expansion. These combined effects contribute to the long-term regulation of atrial pressure, especially during states of hypovolemia or hypotension (49, 50).

### 2.5 Autonomic adaptations

During exercise, descending input from motor and premotor cortices activate the hypothalamus, raising sympathetic outflow and suppressing parasympathetic activity. This allows for increased cardiac output and blood flow redistribution. The baroreflex is transiently reset to permit higher arterial pressures without reflex bradycardia (51, 52). In orthostatic stress, typically caused by gravitational shifts in blood volume (e.g., from standing up quickly), a reduction in baroreceptor stretch occurs due to a drop in arterial pressure. This elicits a rapid sympathetic response resulting in vasoconstriction and tachycardia to maintain blood pressure. However, a paradoxical surge in vagal activity, usually triggered by emotional distress or pain, can override this response causing vasovagal syncope, highlighting the sensitivity of autonomic balance (53, 54).

## 3 Effects of disrupted autonomic cardiovascular control after SCI

SCI interrupts the integrated neural pathways responsible for autonomic cardiovascular regulation, as described above. The cardiovascular consequences that follow injury are primarily due to the loss of supraspinal input to preganglionic neurons in the intermediolateral cell column of the spinal cord (8, 28, 31, 38). After an SCI, particularly at cervical or high thoracic levels (at or above T6), this disconnection creates a clinical state of unopposed parasympathetic vagal influence and a loss of sympathetic control below the lesion (3, 8, 55). The severity of hemodynamic compromise increases with higher and more complete injuries. High level injuries impair the descending sympathetic outflow to vascular beds such as the splanchnic circulation and heart, yielding profound vasodilation and bradycardia. Further, there is a loss of sympathetic vasoconstriction leading to systemic hypotension, often accompanied by reflex bradyarrhythmia due to unopposed vagal activity (27, 56). This is exacerbated by the disrupted arterial baroreflex pathways being unable to provide rapid blood pressure compensation (39). Together, these clinical manifestations of hypotension and bradycardia due to loss of sympathetic tone define neurogenic shock. This is a life-threatening condition that ensues within minutes of injury and in severe cases can persist for several weeks (8, 57).

In the acute phase of SCI, the failure of autonomic cardiovascular control makes the injured spinal cord especially vulnerable to secondary damage as the normal processes that manage perfusion are lost (3, 8). As a result, the secondary injury cascade is transformed from a local spinal process into a systemically driven phenomenon that exacerbates the severity of injury and undermines neurological recovery. Below, the major components of secondary injury are outlined alongside the mechanism(s) by which hemodynamic instability due to autonomic dysregulation impacts each process.

### 3.1 Vascular disruption and ischemia

The loss of sympathetic outflow disrupts the maintenance of perfusion pressure in the spinal cord’s microvasculature, causing it to become solely dependent on systemic pressure and positioning. Without baseline vasomotor tone or reflex vasoconstriction, acute systemic hypotension ensues, producing a critical reduction in blood flow through the injured cord. Animal and human studies have shown that even brief periods of reduced MAP can expand the area of ischemic damage and worsen long-term neurological outcomes (11, 58). It has been emphasized that ischemia is a major contributor to tissue loss, second only to the primary mechanical injury (10).

### 3.2 Blood-spinal cord barrier breakdown

Endothelial cells within the injured cord are sensitive to changes in shear stress, oxygen tension, and pressure. Prolonged hypotension and intermittent hypoxia induces endothelial dysfunction and tight junction disassembly, increasing blood-spinal cord barrier (BSCB) permeability (59). While inadequate perfusion can lead to endothelial cell swelling and/or death, reperfusion surges can physically stretch capillaries and promote barrier leakage. Zhou and colleagues (60) observed that within minutes of injury, abnormal venous pooling and pressure changes occur causing junctional gap formation in capillary endothelium and leakage of the BSCB. Leukocytes are rapidly recruited to these leaky vessels (within 15–30 min post-SCI) and transmigrate through the vessels, further widening the junctional gap and exacerbating BSCB breakdown. Together, these events result in vasogenic edema and accumulation of pro-inflammatory cells at the injury site, enhancing secondary damage (58).

### 3.3 Excitotoxicity and ionic imbalance

Neurons and glia in the injured spinal cord require adequate blood flow to meet their energy demands. The ischemic local environment due to inadequate blood flow leads to rapid depletion of ATP and subsequent failure of energy-dependent ion pumps (61, 62). Consequently, neurons accumulate intracellular sodium and calcium ions, leading to depolarization and release of excess glutamate into the extracellular space. This excess glutamate over activates NMDA and AMPA receptors on local neurons, triggering an excitotoxic process with calcium-mediated cell injury and death.

### 3.4 Oxidative stress

Cycles of ischemia and reperfusion due to hemodynamic instability initiate bursts of oxidative damage after SCI. In an uninjured state with normal autonomic cardiovascular regulation, blood flow is maintained within a narrow range. After SCI, impaired sympathetic regulation leads to a loss of the vasomotor responses that normally dampen extreme fluctuations of blood flow. The combination of baseline hypotension and intermittent hypertensive states (autonomic dysreflexia) brings a wide range of perfusion pressures at the injury site (9, 63). It has been observed that regions of the injured spinal cord experience repeated phases of low perfusion followed by hyperemia in the acute phase of SCI (58). When perfusion is restored to ischemic tissue, a surge of reactive oxygen and nitrogen species is generated (64). These free radicals attack lipids, proteins, and DNA, further increasing cellular injury with each occurrence.

### 3.5 Inflammation

Necrotic cell death in hypoperfused spinal tissue leads to the release of danger-associated molecular patterns (DAMPs), which are not effectively cleared from the site of injury. DAMPs activate pattern recognition receptors on resident microglia/ astrocytes and bolster the recruitment peripheral immune cells (65). Combined with the BSCB disruption, this facilitates the influx of circulating leukocytes, complement, and other inflammatory mediators (66). The resulting prolonged inflammatory response allows for increased local tissue loss and lesion expansion, impeding neurological recovery (67).

Additionally, SCI induces a systemic immunosuppressive state driven by maladaptive sympathetic and hypothalamic activation (68, 69). Disinhibited sympathetic outflow releases excess NE at immune organs such as the spleen and lymph nodes. This contributes to apoptosis of T- and B-cells and reduces cytokine production. Concurrently, activation of the hypothalamic–pituitary–adrenal axis drives systemic glucocorticoid release, further suppressing the inflammatory cascade. Together, this activity leads to dampened peripheral immune processes, even as inflammation persists at the injury site.

### 3.6 Apoptosis and oligodendrocyte loss

Neurons and oligodendrocytes are highly sensitive to ischemic events. Following SCI, repeated bouts of hypoperfusion lead to extended periods of metabolic stress in the spinal tissue surrounding the primary injury. This contributes to widespread apoptosis of both neurons and oligodendrocytes that expands the area of secondary injury. Fluctuating blood pressure in the subacute phase of SCI has been found to correlate with greater oligodendrocyte loss and demyelination (58). As oligodendrocytes die and myelin degenerates, previously functional axons in spared tissue lose conductance, worsening the neurological deficit. Hawryluk et al. (2015) found that patients with fewer periods of hypotension in the acute phase had greater white matter preservation, suggesting that maintaining stable perfusion pressures helps limit apoptosis (11).

### 3.7 Hemorrhage expansion

Immediately after injury, there is often a central area of hemorrhage at the injury site due to ruptured microvessels. Without intact autonomic reflexes, the balance of spinal cord perfusion pressure is lost. Low pressures leads to tissue infarction that then bleed upon reperfusion, while high pressures can further rupture already damaged vasculature with increased blood infiltrating the spinal parenchyma (70). Extravasated blood is cytotoxic to the local spinal tissue. Hemoglobin from ruptured red blood cells breaks down into heme and free iron, which drives oxidative stress and neuronal death via lipid peroxidation and iron-catalyzed hydroxyl radical formation (71, 72). Additionally, heme activates inflammatory signaling via TLR4, promoting cytokine release and lesion expansion (73). Increased hemorrhage at the lesion site is associated with worsened locomotor outcomes in rodents (74–76). This is further supported by human studies, which have shown intramedullary hemorrhage is a strong predictor of irreversible motor paralysis in cervical SCI (77, 78).

## 4 Hemodynamic management in acute SCI

Preventing further spinal cord ischemia during the acute phase of SCI remains one of the few interventions available to clinicians to improve neurological recovery following injury. This involves prompt surgical decompression of the injured spinal cord, coupled with vigilant respiratory management and proactive hemodynamic monitoring and support (79–81). These interventions are critical to minimize secondary ischemic damage allowing for maximal tissue preservation, as discussed above (10, 29, 82). Here, we discuss the evolution of currently available therapeutic interventions as well as future directions for the treatment of hemodynamic instability during the acute phase of SCI.

### 4.1 Targeting MAP

Over the past few decades, research has focused on optimizing hemodynamic protocols that maintain spinal cord perfusion and limit secondary injury after SCI (Table 3). In 2008, the Consortium for Spinal Cord Medicine issued the first formal guidance to maintain MAP at or above 85 mmHg for at least 7 days post injury (83, 84). Interestingly, this recommendation was largely based on the results from two small, uncontrolled clinical studies from the 1990s by Levi et al. (85) and Vale et al. (86). In 2013, the American Association of Neurological Surgeons and the Congress of Neurological Surgeons set a more stringent guideline recommending MAP be maintained between 85 and 90 mmHg for 7 days (87). Because these guidelines were based on limited and low-quality data and were difficult to achieve in routine ICU care, subsequent studies have emphasized three questions: (1) When to initiate MAP augmentation; (2) where to set practical lower and upper MAP bounds; and (3) how long to continue support?

**Table 3:** MAP-targeted strategies in acute SCI.

Source	Design	Injury	Protocol	Follow-up	Primary outcome	Key finding
Levi 1993 (85)	Prospective case series; *N* = 50	Acute cervical SCI; complete and incomplete	Aggressive hemodynamic support with fluids + vasopressors; target MAP > 90 mmHg for 7 days post-SCI	6 weeks	Neurologic improvement (modified Frankel and motor scores)	Protocol feasible and safe; hemodynamic parameters did not distinguish complete versus incomplete injuries, but a “severe hemodynamic deficit” profile (disproportionately low PVRI versus SVRI) predicted no recovery in Frankel A + B patients, whereas 13 of 29 without this profile improved.
Vale 1997 (86)	Prospective series; *N* = 77 (64 with 12-mo follow-up)	Acute cervical and thoracic SCI; ASIA A–D	SBP > 90 and MAP 85–90 mmHg; vasopressors + fluids; typically 7 days; early decompression when feasible	Neurologic outcome at discharge and 12 months	ASIA conversion / motor recovery	MAP augmentation strategy with early decompression associated with higher-than-expected neurological improvement versus historical controls.
Hawryluk 2015 (11)	Observational cohort; *N* = 74	Acute SCI; first 2–3 days post-injury	Exposure analysis of MAP levels (no fixed target); evaluated thresholds including ≥85 mmHg across first 2–3 days	Discharge and 6-month neurologic outcome	Association of MAP exposure with ASIA motor score / conversion	Higher MAP early after injury correlated with better neurologic recovery; hypotension predicted worse outcomes.
Catapano 2016 (94)	Retrospective cohort; *N* = 62	Acute complete SCI at presentation (initial ASIA A)	MAP values measured over first 7 days; vasopressor use captured	Discharge neurologic status	ASIA conversion from complete (A) to higher grade	Higher mean MAP values associated with neurologic improvement; vasopressor requirement tracked with severity.
Haldrup 2020 (14)	Retrospective cohort; *N* = 129	Acute SCI	Initial ED/prehospital MAP levels analyzed	Long-term functional outcome	Association of initial MAP with outcome	Higher initial MAP associated with better long-term outcome; hypotension predicted worse recovery.
Weinberg 2021 (93)	Retrospective single-center cohort; *N* = 136	Acute blunt SCI; mixed ASIA A-D	MAP goal ≥85 mmHg for first 72 h; vasopressors as needed	Hospital discharge	ASIA grade change	Greater proportion of MAP readings ≥85 mmHg predicted ASIA improvement; effect mediated by vasopressor dose.
Clark 2023 (88)	Retrospective cohort; *N* = 99	Acute SCI	Depth of prehospital hypotension and lowest prehospital MAP analyzed versus MRI neuroanatomy	Early ASIA grade / MRI lesion measures	Probability of motor-incomplete injury; neural tissue preservation	Each 5-mmHg increase in lowest per-episode MAP increased odds of motor-incomplete injury; hypotension linked to neural tissue loss.
Torres-Espín 2021 (89)	Retrospective multicenter cohort; *N* = 118 (confirmatory regression *N* = 103)	Acute SCI	Data-driven analysis of intraoperative MAP	Hospital Discharge	ASIA grade change (≥ 1 level)	Identified a target range of approximately 76 to 104–117 mmHg; more time spent outside the optimal MAP range was associated with a lower probability of neurologic improvement.
Chou 2022 (90)	Retrospective multicenter cohort; *N* = 74 training, *N* = 59 external validation	Acute SCI	Modeled intraoperative MAP features based on thresholds identified by Torres-Espín 2021	Hospital Discharge	ASIA grade change (≥ 1 level)	Hypertension during surgery was a stronger negative correlate than hypotension; avoiding MAP above ~104 mmHg improved outcomes.
Almeida 2021 (91)	Multicenter preclinical secondary analysis (MASCIS); *N* = 1,125 rats	Thoracic contusion SCI in rats (T9–10), drop heights 12.5, 25, 50 mm	Perioperative MAP via arterial catheter (20 min pre-injury, at injury, 20 min post-injury); relationship of MAP to recovery assessed	Acute 48 h and weekly to 6 weeks	BBB locomotor recovery change, % weight gain	Interaction between MAP and injury severity: in moderate injury, higher MAP predicted better recovery; in severe injury, higher MAP predicted worse recovery.
Lariccia 2024 (92)	Retrospective single-center cohort; *N* = 53	Acute SCI; ASIA A–D at presentation	Early ‘hyperperfusion’ (mean MAP >90 mmHg within first 8 h and over 72 h) versus standard care	Discharge and/or 6-month outcome	ASIA conversion / motor score improvement	Early sustained MAP >90 mmHg associated with greater neurologic improvement compared with standard care.
Drotleff 2022 (95)	Prospective pilot; *N* = 20	Acute SCI	Protocolized MAP ≥85–90 mmHg with fluids/vasopressors guided by PPV/SVV/CI over 7 days	ICU course; early neurologic exam	Feasibility; hemodynamic optimization metrics	Protocol achieved MAP targets with minimal fluid overload; highlighted utility of advanced monitoring to avoid under/over-resuscitation.
Långsjö 2024 (96)	Retrospective two-cohort comparison; *N* = 51 (higher MAP *n* = 32; lower MAP *n* = 19)	Acute cervical SCI	ICU MAP targets 85–90 versus 65–85 mmHg; 1-min recording over up to 7 days; 3-day MAP % ≥ 85 summarized	ASIA conversion at discharge	ASIA improvement versus MAP target group and MAP % ≥ 85	Higher MAP targets produced greater elevated MAP exposures but did not yield statistically different neurologic recovery; MAP % ≥ 85 did not correlate with outcome.

Clinical and preclinical studies of mean arterial pressure augmentation, including design, injury level, protocol, follow-up, outcomes and key findings. MAP, mean arterial pressure; SCI, spinal cord injury; ASIA, American Spinal Injury Association Impairment Scale; SBP, systolic blood pressure; MRI, magnetic resonance imaging; BBB, Basso–Beattie–Bresnahan locomotor score; PVRI, pulmonary vascular resistance index; SVRI, systemic vascular resistance index; PPV, pulse pressure variation; SVV, stroke volume variation; CI, cardiac index; ICU, intensive care unit; ED, emergency department.

#### 4.1.1 Initiation timing

Hawryluk et al. (11) provided early evidence that higher MAP values in the first 2–3 days post injury correlate with greater neurological improvement, whereas prolonged hypotension correlated with worse outcomes. In their analysis of 100 patients who received 5 days of MAP targeted therapy (>85 mmHg), they found the proportion of time below the 85 mmHg threshold during the initial 72 h was a stronger predictor of poor recovery than average MAP. This suggests that preventing hypotension may be more critical than achieving supraphysiologic pressures. Building on this, Haldrup et al. (14) demonstrated that maintaining MAP above 80 mmHg as early as pre-hospital transport through the intraoperative and ICU periods was associated with significantly better long-term motor outcomes 1-year following injury in a retrospective cohort of 129 patients. Consistent with these findings, Clark et al. (88) reported that even brief hypotension prior to hospital arrival can exacerbate secondary injury. In their sample of 99 SCI cases, each 5 mmHg increase in the lowest recorded prehospital MAP was associated with a 34% increase in the likelihood of an incomplete (versus complete) neurological grade at admission. These findings underscore the importance of early and aggressive blood pressure support immediately after SCI, even before patients reach the hospital.

#### 4.1.2 Double edged sword of MAP limits

While avoiding hypotension is clearly vital, emerging data also highlight the potential risks of excessive hypertension (i.e., hyperperfusion). Torres-Espin et al. (89) applied machine learning techniques to intraoperative hemodynamic records from 118 acute SCI surgeries across two centers. They identified an intraoperative MAP range with a lower limit of 76 mmHg and an upper boundary between 104 and 117 mmHg that was associated with the best chance of improved neurological recovery. Patients who experienced MAP values outside of this window had significantly worse neurological outcomes, indicating that both hypoperfusion and extreme hyperperfusion during surgery can be detrimental. A follow-up study by Chou et al. (90) corroborated these thresholds using a decision-tree machine learning model. In 74 surgical patients, they found that an average intraoperative MAP of 80–96 mmHg was associated with the highest likelihood of motor grade improvement, whereas all patients whose mean intraoperative MAP exceeded ~96 mmHg showed no neurological improvement. Additionally, spending more than 93 min outside of the 76–104 mmHg range (especially above the upper bound) was linked to worse functional outcomes at discharge. Collectively, these results provide strong evidence that there is an optimal MAP in the acute phase and that both prolonged hypotension and excessive hypertension beyond the upper limit can worsen recovery. Notably, this principle appears to hold in preclinical models as well. A meta-analysis of 1,125 rats with SCI found that low blood pressure exacerbated injury in moderate contusions, whereas high blood pressure worsened outcomes in severe contusions (91).

#### 4.1.3 Hyperperfusion is detrimental

Many centers have historically overshot MAP targets (i.e., induced systemic hypertension) to ensure patients remain above the recommended minimum, but this practice is now being reexamined. One observational study of ICU patients with acute SCI found that maintaining MAP strictly within the 85–90 mmHg guideline was practically unachievable, with a success rate of only about 24% of recorded MAP values lying within this range over the first 5 days (21). Attempts to maintain higher mean pressures tended to increase MAP variability and expose patients to more episodes of hypertension. Nonetheless, some have postulated that deliberately tolerating or inducing hyperperfusion might confer additional benefit to the injured cord (92). In a single-center retrospective cohort, early MAP goal attainment correlated with better short-term neurological improvement: patients who spent less time below 85 mmHg in the first 12 h had higher odds of ASIA grade improvement, and their mean MAP was higher during that window (92). Notably, the cohort with early improvement exhibited incomplete injuries (ASIA C) at baseline and far fewer interfacility transfers, though the analysis lacked multivariable adjustment. These factors warrant caution in inferring causality and suggest that early target attainment may be, in part, a marker of baseline neurological status and care pathway rather than a pure effect of hyperperfusion alone. Their observations reinforce other findings that more time spent with blood pressure above 85 mmHg early after injury is associated with improved neurological outcomes (93, 94). However, there is very limited evidence to support pushing MAP to higher values that may tax the physiologic limits of the spinal cord. The potential downsides of vasopressor-induced hypertension (cardiac stress, arrhythmias, peripheral ischemia, etc.) have led many investigators to question the common practice of routinely overshooting MAP targets (21, 95). A pilot study by Drotleff et al. (95) using advanced hemodynamic monitoring found that acute SCI patients often have a reduced systemic vascular resistance index (due to lack of sympathetic outflow to blood vessels) while MAP, cardiac index, and heart rate were in reference ranges. Thus, they argue that vasopressor use to achieve very high MAP levels may not bolster spinal cord perfusion.

#### 4.1.4 Lower MAP targets

On the other end of the spectrum, studies have explored whether lower MAP goals might be sufficient for improved outcomes. Långsjö et al. (96), performed a retrospective comparison of two MAP protocols in 51 patients with cervical SCI for at least 3 days post injury: One group was managed to a traditional MAP of 85 to 90 mmHg in the ICU, while the other had a more moderate target of 65 to 85 mmHg. As expected, the higher MAP group received more vasopressors and achieved substantially higher pressures. Importantly, however, neurological recovery at 1 year was equivalent between the two groups, and there was no correlation between individual MAP levels and degree of motor improvement. These clinical findings suggest that a more modest MAP target may be adequate. This work aligns with the earlier observations from Martin et al. (97) finding that augmenting blood pressure to meet MAP goals of 85 to 90 mmHg in the first 72 h conferred no significant improvement in ASIA motor scores by hospital discharge. In their cohort, patients with more severe injuries had more hypotensive episodes and consequent vasopressor treatment to maintain MAP, yet the blood pressure augmentation itself did not translate into superior recovery. Together, these studies suggest that for many patients, preventing hypotension with a practical MAP floor near 75 to 80 mmHg may be sufficient, while enforcing a narrow supraphysiologic target offers no proven neurologic advantage.

#### 4.1.5 Impact on guidelines

Evolving insights from the past decade have informed updates to clinical guidelines. Most recently, in 2024 a new multidisciplinary guideline for hemodynamic management in acute SCI by AO Spine, American Association of Neurological Surgeons and the Congress of Neurological Surgeons was published (22). They recommended augmenting MAP to at least 75 to 80 with an upper limit not to exceed 90 to 95 mmHg. The guideline also reduced the suggested duration of MAP support to 3–7 days after injury, acknowledging the most critical period for perfusion is likely within the first several days. Notably, the panel graded the evidence as very low and the recommendations as weak, emphasizing the need for an individualized approach tailored to each patient. These recommendations align with AO Spine guidance on decompression timing, which favors surgery within 24 h when medically feasible and underscores the need to individualize timing based on patient and injury characteristics (25). The accompanying AO Spine Clinical practice recommendations describe contemporary surgical strategies and emphasize that perfusion targets, decompression, and postoperative ICU management are most effective when implemented as a coordinated acute care pathway (26). Taken together, these hemodynamic and surgical updates represent a more nuanced approach that balances the need to prevent hypotension against the risks of excessive hypertension in the early post-injury period.

### 4.2 Targeting spinal cord perfusion pressure

After SCI, the injured cord often swells within the spinal canal, raising intrathecal pressure and reducing spinal perfusion even when systemic MAP is normal (98). Thus, augmen7ng blood pressure while u7lizing MAP as a therapeu7c target alone may be insufficient. Due to this, utilizing spinal cord perfusion pressure (SCPP) as a target has emerged as a promising and more reliable hemodynamic strategy in acute SCI. SCPP represents the pressure gradient driving blood flow through the injured spinal cord, defined as MAP minus intrathecal (or intraspinal) pressure. This is analogous to cerebral perfusion pressure management in traumatic brain injury, where intracranial pressure is subtracted from MAP to ensure adequate brain perfusion (99). Intrathecal pressure (ITP) and intraspinal pressure (ISP) are two terms (defined below) that are sometimes inaccurately used interchangeably when describing SCPP-guided therapy. Obtaining ITP and ISP measures to calculate SCPP requires different monitoring techniques, each with their own safety, efficacy, and feasibility concerns. There are limited data on the feasibility, safety, and effects of targeting SCPP as compared to MAP (19, 100). This remains an active area of research with early results reviewed here (Table 4).

**Table 4:** SCPP-targeted monitoring and management in acute SCI.

Source	Design	Injury	Monitor / device	Protocol	Follow-up	Primary outcome	Key finding
Kwon 2009 (101)	RCT; *N* = 22	Acute cervical SCI; ASIA not specified	ITP via lumbar intrathecal catheter + arterial line	Drainage for 72 h to reduce ITP and raise SCPP; MAP augmented per ICU protocol	72 h and 6-month	Feasibility and effect on SCPP	CSF drainage increased SCPP at 72 h; no between-group difference in 6-month motor outcomes.
Werndle 2014 (98)	Prospective feasibility cohort; *N* = 18	Acute cervical and thoracic SCI; ASIA A–C	ISP at injury site via subdural Codman microsensor	Probe inserted ≤72 h post-injury; monitored up to 1 week. Observed effect of dural decompression	ICU monitoring ≤ 1 week	Feasibility/safety; spinal cord physiology	High ISP and low SCPP common after laminectomy; dural decompression improved SCPP and reactivity (sPRx).
Varsos 2015 (107)	Observational cohort; *N* = 18	Acute cervical and thoracic SCI; ASIA A–D	ISP probe at injury site + arterial line	Probe within 72 h; monitored up to 1 week; waveform and reactivity analysis	ICU monitoring ≤ 1 week	Biophysics of ISP/SCPP; sPRx relation to SCPP	Identified U-shaped sPRx–SCPP curve and pressure–volume features; foundational for SCPPopt concept.
Phang 2015 (106)	Prospective cohort; *N* = 21	Acute cervical SCI; ASIA A–C	ISP probe at injury site	Monitored up to 1 week; compared bony decompression versus expansion duroplasty	2–3 weeks MRI; 6-month outcomes	Radiologic decompression and spinal cord physiology	Duroplasty lowered ISP, increased SCPP, and improved sPRx; group mean SCPPopt 90 mmHg (patient range 60–120 mmHg).
Chen 2017 (108)	Prospective cohort; *N* = 14	Acute cervical and thoracic SCI; ASIA A–C	Injury-site ISP probe (subdural Codman) + arterial line; continuous SCPPopt calculation; microdialysis in subset	Continuous SCPP monitoring in ICU; individualized SCPPopt derived; no lumbar drain	ICU monitoring ≤ 1 week; 2 wk.; 6–12 month outcomes	Feasibility of continuous SCPPopt visualization	Continuous determination of patient-specific SCPPopt feasible; supports individualized hemodynamic targets.
Squair 2017 (102)	Prospective cohort; *N* = 93	Acute cervical and thoracic SCI; ASIA A–C	Lumbar intrathecal catheter (no CSF drainage)	MAP targeted (80–85 mmHg) 7 days; SCPP monitored	Discharge and 6-month outcomes	Association of SCPP with neurological recovery	Augmented SCPP associated with better motor recovery; evidence that perfusion matters clinically.
Squair 2019 (103)	Retrospective cohort; *N* = 107	Acute cervical and thoracic SCI; ASIA A-D	ITP via lumbar catheter	Analyzed exposure to SCPP levels over first week	6-month outcomes	Empirical SCPP target windows	SCPP 60–65 mmHg associated with ASIA improvement; extremes above/below associated with worse outcomes.
Hogg 2020 (105)	Prospective cohort; *N* = 13	Acute SCI; cervical, thoracic, and conus; ASIA A–C	Concurrent lumbar CSF (ITP) catheter and injury-site ISP probe; microdialysis in subset	Concurrent ISP and lumbar CSF monitoring; intermittent CSF drainage to test ISP effects; lumbar CSF metabolites versus injury-site micro dialysate.	ICU monitoring ≤ 1 week; MRI at 2–3 months; 6 month outcomes	Correlation between lumbar CSF and injury-site ISP–derived SCPP; Effect of CSF drainage on ISP	Lumbar CSF pressure poorly reflected injury-site ISP and SCPP; lumbar drainage rarely reduced ISP; injury-site microdialysis values differed from lumbar CSF metabolites.
Yue 2020 (15)	Prospective Cohort; *N* = 15	Acute cervical and thoracic SCI; ASIA A-D	Lumbar subarachnoid drain (LSAD)	LSAD within 24 h; SCPP monitored 5 days goal ≥65 mmHg; MAP not targeted separately; early surgical decompression in 14/15 patients	ICU monitoring for 5 days; ASIA at day 7 versus admission	Feasibility and safety of standardized SCPP-guided protocol; ASIA change	SCPP protocol was feasible and safe; all patients maintained mean SCPP ≥65 mmHg; no LSAD-related complications; at day 7, 33% overall and 50% of severe ASIA A–C patients improved ≥1 ASIA grade.

Studies utilizing intrathecal (ITP) or intraspinal (ISP) pressure monitoring to calculate spinal cord perfusion (SCPP) pressure with associated protocols, outcomes, and key findings. SCPP, spinal cord perfusion pressure; SCPPopt, optimized spinal cord perfusion pressure; MAP, mean arterial pressure; ISP, intraspinal pressure; ITP, intrathecal pressure; sPRx, spinal pressure reactivity index; ICU, intensive care unit; ASIA, American Spinal Injury Association Impairment Scale; MRI, magnetic resonance imaging; CSF, cerebrospinal fluid; LSAD, lumbar subarachnoid drain.

#### 4.2.1 Monitoring intrathecal pressure as a measure of SCPP

ITP reflects the pressure of the CSF within the thecal sac (the subarachnoid space around the spinal cord). In acute SCI, cord swelling and impaired CSF circulation can elevate ITP, reducing SCPP and further worsening recovery. Monitoring and titrating ITP using a lumbar intrathecal catheter has been found to be safe and feasible in the acute setting (101). In a prospective trial, Kwon et al. (101) inserted lumbar CSF catheters in 22 patients within 48 h of SCI for continuous ITP monitoring with half receiving CSF drainage. They observed that the mean ITP was approximately 14 mmHg at insertion, rising to about 22 mmHg during surgical decompression, and often further increased post-operatively. Importantly, draining CSF dampened these ITP elevations without any significant complications. This study established proof-of-concept that lowering ITP via lumbar drainage can effectively improve SCPP in acute SCI and can be implemented with low risk. Subsequent studies have reinforced that SCPP is more predictive of neurologic recovery than MAP alone, further supporting the value of ITP monitoring (15, 102). One study analyzed 92 acute SCI patients with lumbar CSF monitors and found that time spent above an SCPP of 50 mmHg had a positive linear relationship with 6-month motor improvement (102). Further work showed that maintaining SCPP in the 60 to 65 mmHg range was associated with the greatest neurological improvement, whereas adherence to MAP targets was less predictive (103). Preclinical data support these ranges by showing that augmenting perfusion improved spinal cord blood flow and metabolism, but with diminishing returns and potential harm at excessive pressures depending on the vasopressor used (104). Further, Yue and colleagues (15) successfully implemented the use of a SCPP-guided therapy protocol utilizing a lumbar drain to monitor ITP. In their study of 15 acute SCI patients, they targeted a SCPP at or above 65 mmHg for 5 days instead of traditional MAP goals. No drain related complications occurred and one-third of patients showed improved ASIA grades by day 7, further suggesting an SCPP-guided approach is clinically feasible and may confer early neurological benefits. Together, these studies demonstrate the clinical utility of employing lumbar CSF catheters to monitor and augment SCPP to promote recovery after SCI with the potential to lessen the use of vasopressors. A key limitation of lumbar catheters is that they measure CSF pressure distal to the injury, which may underestimate true pressures at the lesion site in cases of severe spinal cord swelling from hemorrhage or edema (98, 99, 105). Nonetheless, monitoring ITP with lumbar catheters is generally safe, feasibly integrated into clinical settings, and provides insight into cord perfusion status that might be missed when solely relying on MAP monitoring (19).

#### 4.2.2 Monitoring intraspinal pressure as a measure of SCPP

ISP refers to the pressure within the intradural space at the injury site measured via a probe placed under the dura, typically after surgical decompression. This technique was pioneered by the Injured Spinal Cord Pressure Evaluation (ISCoPE) studies and overcomes the limitation of ITP monitoring, which sometimes underestimates pressures at the site of injury (98, 99). In addition to providing proof-of-principle of this protocol, the work by Werndle and colleagues (98) showed that surgical decompression via laminectomy alone did not reliably reduce ISP and may even be detrimental by exposing the swollen spinal cord to further external compression forces. This finding motivated a subsequent study evaluating the use of expansion duroplasty (enlarging the dural sac) to further decrease ISP (106). Performing an expansion duroplasty in 10 patients after laminectomy decreased ISP and increased SCPP. This is just one example of how direct measurement of ISP can guide decisions in real time. Moreover, continuous ISP monitoring has enabled advanced insights into spinal cord hemodynamics (107). By concurrently recording MAP and ISP, investigators can calculate SCPP and spinal pressure reactivity index (sPRx) on a rolling basis, analogous to intracranial pressure monitoring in TBI (107). The sPRx is derived as the correlation between slow waves of ISP and MAP, where a positive correlation indicates impaired spinal blood flow autoregulation and a negative or zero relation suggests intact autoregulation. Using these techniques, Chen and colleagues (108) identified an optimal SCPP for each patient by finding the pressure that minimized sPRx. In their series of 45 patients with ISP monitors, the continuous optimal SCPP could be calculated about 45% of the time and was highly variable between individuals (108). Even within a single patient, the optimal SCPP shifted over hours to days, demonstrating a dynamic perfusion requirement. Importantly, time spent out of a patient’s optimal SCPP was associated with a decline in key metabolic markers and worsened neurologic outcomes. Patients that stayed within an average of 5 mmHg of their optimal SCPP had significantly greater ASIA grade improvement compared to those who did not. These findings provide compelling evidence that individualized SCPP goals, made possible using ISP monitoring at the injury site, can optimize neurological outcomes. Major limitations of ISP monitoring include its invasive nature requiring neurosurgical expertise with risk of further injury, specialized hardware, and continuous data capture and analysis, all of which limit its availability outside specialized centers (109, 110). Still, targeting ISP provides the most direct measure of the local pressure at the level of injury that has allowed for valuable insight advancing the use of SCPP-guided therapy in acute SCI.

#### 4.2.3 Future of SCPP

Despite the advancements made in the use of targeting SCPP in acute SCI, detailed studies to guide its clinical implementation are still needed. Ongoing and upcoming clinical trials are poised to provide much needed data. A randomized controlled trial at UTHealth Houston has been launched to compare traditional MAP management versus SCPP-targeted care in acute SCI (“Monitoring spinal cord perfusion pressure in acute traumatic spinal cord injury”; Clinicaltrials.gov NCT06451133). This trial will be one of the first to test whether proactive SCPP optimization translates into superior recovery compared to MAP-driven care, and will also clarify the risks associated with each strategy. Complementing this, the Canadian-American SCPP and Biomarker Study (“CASPER”; Clinicaltrials.gov NCT03911492) is an ongoing multicenter trial investigating the effect of SCPP versus MAP on long-term outcomes. Both of these studies are utilizing lumbar catheterization to monitor and manipulate ITP to optimize SCPP. Together, this work will help determine whether ITP monitoring and SCPP-targeted treatment should become the standard of care and will guide future work in optimizing MAP and SCPP protocols.

### 4.3 Vasopressor selection

The immediate cardiovascular consequence of SCI is hypotension, and for this reason, targeting MAP and SCPP goals involves treatments designed to augment blood pressure, with initial management involving IV fluid resuscitation. When a patient remains hypotensive despite adequate fluid resuscitation, vasopressor therapy is initiated to augment systemic vascular resistance and raise blood pressure into the targeted MAP range (111). Vasopressors serve as pharmacological support to counteract the loss of sympathetic tone and maintain adequate spinal cord perfusion. Several vasopressors are available, but three are most commonly utilized in acute SCI: NE, dopamine (DA), and phenylephrine (PHE) (12, 79, 81, 112) (Table 5). Each agent has a distinct mechanism of action and hemodynamic profile, which informs its use in different clinical scenarios. Although all can effectively raise MAP, their differing affinities for *α*- and *β*-adrenergic (and dopaminergic) receptors lead them to exert unique effects on heart rate, cardiac output, and vascular tone (41, 113, 114). Further, these pharmacological differences translate to variable impacts on spinal cord perfusion and potential side effects. Thus, selecting an appropriate vasopressor requires balancing the desired hemodynamic effect with the drug’s risk profile, with particular attention to the possibility that treatment could have a harmful effect by driving MAP/SCPP outside of the optimal range (Table 6). Below, we compare NE, DA, and PHE in the context of acute SCI, focusing on their mechanisms, indications, outcomes, and complications.

**Table 5:** Common vasopressors used to augment MAP in acute SCI.

Agent	Receptor profile	Hemodynamic action (MAP and CO)	Clinical notes	Sources
Norepinephrine	α1-dominant with β1 activity; minimal β2.	↑MAP via ↑SVR; CO neutral to ↑ (β1). In acute SCI, maintains MAP with lower ISP and higher SCPP than dopamine.	Animal and clinical data: norepinephrine improved SCBF/PO_2_ versus phenylephrine post-decompression in pigs; early post-injury rat infusion increased hemorrhage size; lower arrhythmia burden versus dopamine in ICU shock trials.	Altaf 2017 (120); Soubeyrand 2014 (62); Streijger 2018 (119); Cheung 2020 (70); De Backer 2010 (114); De Backer 2012 (113)
Phenylephrine	Selective α1 agonist.	↑MAP via ↑SVR; CO may ↓ due to ↑afterload and reflex bradycardia.	Porcine SCI: lower SCBF and PO_2_ after decompression and greater hemorrhage than control; generally chosen when tachyarrhythmia precludes β1 effects.	Streijger 2018 (119); Ko 2024 (128)
Dopamine	Dose-dependent: D1/D2 (1–3 μg/kg/min) → vasodilation; β1 (3–10) → ↑HR/contractility; α1 (>10) → vasoconstriction.	At moderate doses ↑CO; at high doses ↑MAP. In acute SCI cross-over, higher ISP and lower SCPP versus norepinephrine at similar MAP.	Higher risk of arrhythmias and worse outcomes versus norepinephrine in shock RCTs/meta-analyses; may be reserved when strong inotropy is required and arrhythmia risk acceptable.	Altaf 2017 (120); Readdy 2015 (124); Inoue 2014 (112); De Backer 2010 (114); De Backer 2012 (113); Agarwal 2023 (125)

Receptor profile, hemodynamic effects, and clinical considerations of norepinephrine, phenylephrine, and dopamine in acute spinal cord injury. MAP, mean arterial pressure; SCI, spinal cord injury; CO, cardiac output; SVR, systemic vascular resistance; SCBF, spinal cord blood flow; PO₂, oxygen tension; LOS, length of stay; RCT, randomized controlled trial; HR, heart rate.

**Table 6:** Vasopressor agents in acute SCI.

Source	Model	Design	Vasopressor(s)	MAP protocol	Primary outcome	Perfusion effect	Hemorrhage	Safety profile
Soubeyrand 2014 (62)	Rat T9 contusion	Preclinical; randomized; *N* = 35	Norepinephrine	Dose infusion (no MAP target reported)	SCBF measured by laser Doppler; microvascular flow near lesion edge	NE transiently increased SCBF proximally but was not sustained	Increased parenchymal hemorrhage versus control	Suggests early NE can worsen hemorrhage
Streijger 2018 (119)	Porcine T10 contusion-compression	Randomized; *N* = 22	Norepinephrine versus phenylephrine	Augment MAP by 20 mmHg above baseline	Spinal cord RBC flux and PbtO2	NE increased RBC flux and PbtO2; PE decreased microvascular flow despite MAP ↑	Not reported	Adverse systemic effects not assessed
Cheung 2020 (70)	Porcine thoracic contusion	Preclinical; *N* = 16	Norepinephrine	MAP titrated (~50 → 110 mmHg) post-injury	SCBF (RBC flux) and microvascular oxygenation	SCBF and oxygenation rose linearly with MAP up to ~100–110 mmHg; regional heterogeneity noted	Not increased during step-ups	NE infusion improved perfusion but increased hemorrhage risk in some conditions.
Altaf 2017 (120)	Human acute SCI ICU	Prospective cross-over; *N* = 11	Norepinephrine versus dopamine	Clinical MAP maintenance ≥85–90 mmHg	SCPP via lumbar subarachnoid catheter	NE increased SCPP and reduced ISP versus dopamine	Not reported	DA caused tachycardia → arm terminated; NE favored for microcirculation
Readdy 2015 (124)	Human ATCCS	Retrospective cohort; *N* = 34	Dopamine versus phenylephrine	Maintain MAP ≥85 mmHg for up to 7 days	Clinical outcomes; complications	Not applicable	Not reported	Dopamine associated with more CV complications versus phenylephrine in older patients
Readdy 2016 (126)	Human SCI (penetrating versus blunt)	Retrospective cohort; *N* = 36	Dopamine and phenylephrine	Maintain MAP ≥85 mmHg; mean ~101–124 h exposure	Complications by agent/mechanism	Not applicable	Not reported	Dopamine linked to more cardiogenic complications; phenylephrine fewer events
Agarwal 2023 (125)	Elderly (≥ 65 years) human SCI	Prospective registry; *N* = 40	NE, PE, VP, DA, Dob, Epi	Maintain MAP ≥85 mmHg	Complications, LOS, mortality	Not applicable	Not reported	Norepinephrine use associated with more CV complications; caution in elderly
Ko 2024 (128)	Rat cervical SCI	Preclinical randomized; *N* = 48	Norepinephrine and phenylephrine	Pressor infusion to raise MAP; decompression timing examined	Intraspinal pressure, microvascular leakage	Pressors increased MAP; but exacerbated microvascular extravasation without decompression	Decompression reduced vasopressor induced spinal hemorrhage and extravasation	Pair hemodynamic therapy with decompression to mitigate bleeding risk
Hashemaghaie 2025 (127)	Human acute SCI	Retrospective cohort; *N* = 277	Norepinephrine; dopamine, and phenylephrine	MAP ≥85 mmHg; early versus non-early pressor initiation compared	Clinical outcomes; complications; LOS	Not applicable	Not reported	Early pressor use not clearly associated with improved outcomes; cardiovascular events frequent

Preclinical and clinical studies examining vasopressor use, hemodynamic protocols, perfusion effects, and safety outcomes. MAP, mean arterial pressure; SCI, spinal cord injury; SCBF, spinal cord blood flow; PbtO₂, partial pressure of tissue oxygen; ISP, intraspinal pressure; SCPP, spinal cord perfusion pressure; NE, norepinephrine; PE, phenylephrine; DA, dopamine; Dob, dobutamine; Epi, epinephrine; VP, vasopressin; ATCCS, acute traumatic central cord syndrome; CV, cardiovascular; RBC, red blood cell.

#### 4.3.1 Norepinephrine

NE is widely considered the first-line vasopressor for acute SCI due to its balanced adrenergic activity and favorable safety profile (14, 15, 111, 112). NE is a potent α1 adrenergic agonist with modest β1-adrenergic effects. This results in strong peripheral vasoconstriction, with a mild increase in heart rate and contractility (115, 116). In the setting of SCI-induced vasodilation and bradycardia, NE effectively counteracts hypotension while providing inotropic support, a useful combination in neurogenic shock (79, 81). Across comparative literature, NE is associated with fewer tachyarrhythmias and myocardial complications than DA in shock states, supporting its preference (81, 113, 114, 117, 118). Preclinical SCI work also suggests NE improves spinal cord blood flow and tissue oxygenation more than PHE at equivalent pressure targets, supporting a physiological advantage (119). In addition, NE was able to maintain target MAP levels with a lower ITP and a correspondingly higher SCPP when compared with DA in a study of 11 SCI patients (120). However, there have also been some detrimental effects of NE use in preclinical studies. Early NE treatment during persistent cord compression was also found to increase intraparenchymal hemorrhage despite improving tissue oxygenation in a porcine SCI model (70). This highlights the importance of the timing of vasopressor initiation in relation to decompression. Another preclinical study in rats reported NE increased spinal hemorrhage without improving perfusion (62). Nonetheless, NE provides effective MAP support with relatively fewer cardiac side effects, solidifying its role as the preferred vasopressor in acute SCI (81). Yet, it still requires careful titration and monitoring. Caution is also advised in patients with peripheral vascular disease, as NE’s intense peripheral vasoconstriction can compromise distal circulation, but in most SCI scenarios the benefits of improved perfusion outweigh this concern (116, 121).

#### 4.3.2 Dopamine

DA is an endogenous catecholamine with dose-dependent receptor effects (41, 116, 122): Low doses (≤ 5 ug/kg per min) stimulate D1/D2 dopaminergic receptors in the renal and mesenteric beds causing vasodilation resulting in reduced systemic vascular resistance. At intermediate doses (5–10 ug/kg per min), DA predominantly engages β1 adrenergic receptors increasing heart rate and contractility. Using high doses (> 10 ug/kg per min) activates α1 adrenergic receptors producing systemic vasoconstriction. In the acute SCI and other shock states, low-dose DA can be counterproductive by lowering systemic vascular resistance, while higher doses required to maintain MAP are consistently associated with tachycardia and arrhythmias (113, 114, 118, 123). Historically, DA was widely considered the first line in MAP maintenance after SCI. However, mounting evidence underscoring its higher risk of adverse effects compared to PHE and NE has reduced its use, especially in older populations (90, 112, 124, 125) and those with penetrating trauma (126).

#### 4.3.3 Phenylephrine

PHE is a selective α1 adrenergic agonist that increases systemic vascular resistance and arterial pressure with negligible direct *β*1 stimulation. Reflex bradycardia is common, and the resulting rise in afterload without inotropy can reduce stroke volume and cardiac output in susceptible patients (41, 116). These properties make PHE attractive when β-agonism is undesirable, for example in patients with tachyarrhythmias or myocardial ischemia. Yet, caution is warranted for those with baseline bradycardia, conduction disease, or marginal cardiac output. Several retrospective SCI cohort studies indicate PHE produced fewer cardiogenic complications when compared to DA (112, 124, 126). These data support favoring PHE over DA in most cases. A single clinical study has been performed comparing NE to PHE outcomes in acute SCI (127), which reports NE was more likely to lead to adverse effects compared to PHE. However, NE was more frequently selected in patients with severe injuries, possibly confounding their results. Still, this highlights the need for further investigation. In a preclinical porcine SCI model, PHE was found to increase intraparenchymal hemorrhage relative to controls receiving no vasopressor support (119). Rodent studies reinforce the importance of timing vasopressor initiation relative to decompression surgery as discussed in the NE section. Initiating PHE within minutes of cervical injury increased hemorrhage and BSCB extravasation, whereas decompression mitigated this effect (128). More clinical work is necessary to definitively recommend NE over PHE, but for now, evidence favors NE as first line to support spinal cord perfusion.

#### 4.3.4 Vasopressor limitations

Overall, it must be emphasized that vasopressor therapy, even when successful in achieving MAP targets, has inherent limitations. Vasopressor use, especially when reaching supraphysiological levels of blood pressure, contributes to exacerbated secondary injury processes that can undermine recovery (62, 70, 74, 89, 91, 95, 119, 128). Further, the adverse effects due to the pharmacological profile of the vasopressors themselves can be detrimental and even lethal. While vasopressors remain essential in acute SCI care to manage hemodynamic instability, their use is a double-edged sword. The challenge of preserving spinal cord perfusion without exacerbating injury motivates the search for alternative therapeutic strategies. The use of lumbar catheters to drain CSF to lower ITP and increase SCPP shows promise (15, 101).

### 4.4 Neuromodulatory interventions for hemodynamic control

Conventional hemodynamic management in acute SCI primarily treats the manifestations of a disrupted autonomic system with vasopressors and related measures, rather than restoring autonomic function itself. Neuromodulation offers an alternative that aims to re-engage disrupted spinal circuitry by electrically recruiting somatoautonomic pathways below the lesion to restore autonomic output (129, 130). This has been well documented to be effective in chronic SCI with much work focusing on improving volitional limb movements and recovery of motor functions (131–136). More recently, several groups have applied this principle to treat cardiovascular dysfunction and maintain hemodynamic stability in chronic SCI patients with beneficial effects persisting even after cessation of treatment (137, 138) (Table 7). This early evidence justifies further investigation into neuromodulatory techniques as a potential therapeutic strategy to manage hemodynamic instability in acute SCI with the aim of reducing vasopressor dependence and mitigating long-term cardiovascular deterioration by restoring autonomic regulation. Here, we review both invasive and non-invasive neuromodulatory techniques for acute SCI hemodynamic management from preclinical models and emerging clinical studies.

**Table 7:** Neuromodulation strategies that modulate arterial pressure after SCI.

Source	Design	Technique	Population	Protocol	Timeline	Primary outcome	Key finding
Phillips 2018 (148)	Prospective case series; *N* = 5	Open-loop tSCS at T7–T8	Chronic cervical and thoracic SCI; ASIA A/B	Acute stimulation during head-up tilt testing; 30 Hz, 1 ms biphasic pulses; current titrated 10–70 mA	Single-session tilt testing	Beat-by-beat BP, dP/dt, cerebral blood flow, symptoms	BP, cardiac contractility, and cerebral blood flow normalized within ~60 s; symptoms resolved; absence of leg EMG supported sympathetic recruitment.
Harkema 2018 (138)	Prospective case series; *N* = 4	Open-loop epidural SCS (lumbosacral paddle); CV-targeted programs (CV-scES)	Chronic cervical SCI	Configurations chosen to raise MAP into 110–120 mmHg without motor activation	Repeated sessions over several weeks	MAP, SBP/DBP, HR stability across sessions	Repeated CV-scES sessions improved BP stability and introduced mapping for cardiovascular targets.
Squair 2021 (140)	Prospective first-in-human proof-of-concept; *N* = 4	Closed-loop epidural SCS with beat-to-beat MAP feedback	Chronic high-level SCI	Controller adjusts stimulation of T10–T12 posterior roots to maintain user-defined MAP during orthostasis and AD	Acute repeated sessions	MAP control during tilt and AD provocation; sympathetic microneurography; plasma norepinephrine	Closed-loop control maintained MAP in real time, prevented BP collapse during tilt, and increased sympathetic activity/norepinephrine.
Peters 2023 (145)	Prospective protocol development study; *N* = 10	Systematic tSCS mapping (T7/8, T9/10, T11/12, L1/2)	Chronic SCI with low seated SBP	30 Hz, 1 ms pulse width, 5 kHz carrier; amplitude increased in 5 mA steps; Standardized SBP targets set at 110–120 mmHg (men) or 100–120 (women)	Single-session mapping visit	Feasibility and safety; SBP change; symptom tracking	Mapping identified reproducible, individualized tSCS parameters for BP stabilization.
Engel-Haber 2024 (149)	Prospective case series; *N* = 8	tSCS mapping across cervical to sacral sites	Chronic cervical SCI with BP instability	30 Hz, 1 ms pulse width, 5 kHz carrier; 5 mA step-ups to ~120 mA; compare vertebral levels	Single-session mapping visit	Beat-to-beat change in SBP (calibrated to brachial), DBP, and HR	Lumbosacral (L1/2–S1/2; incl. T11/12) stimulation consistently elevated SBP, whereas cervical/upper thoracic did not, supporting caudal targeting
Hofstoetter 2018 (147)	Mechanistic study; *N* = 20 (prospective tSCS, retrospective eSCS)	tSCS versus eSCS	Chronic SCI; motor-incomplete and complete	Evoke posterior-root muscle reflexes; tSCS via paravertebral electrodes (30 Hz, 1 ms, 5 kHz); eSCS via percutaneous lumbar leads; responses compared with EMG	Single-session testing	Reflex latencies, recruitment curves, and post-activation depression of posterior-root muscle (PRM) reflexes	Both tSCS and eSCS activated common posterior-root afferents; near-identical EMG waveforms confirmed mechanistic overlap.
Shackleton 2023 (146)	Prospective RCT protocol; *N* = 12 planned	Neuromodulation + intensive exercise versus exercise alone	Chronic SCI	Program includes neuromodulation sessions targeting motor and autonomic function	8-week training program	Composite motor outcomes; autonomic outcomes (BP stability) as secondary	Defines trial framework to test combined neuromodulation and exercise effects on BP stability.

Prospective and mechanistic studies using transcutaneous (tSCS) or epidural (eSCS) stimulation approaches to stabilize blood pressure in individuals with SCI. tSCS, transcutaneous spinal cord stimulation; eSCS, epidural spinal cord stimulation; CV-scES, cardiovascular-targeted epidural spinal cord stimulation; AD, autonomic dysreflexia; MAP, mean arterial pressure; SBP, systolic blood pressure; DBP, diastolic blood pressure; HR, heart rate; ASIA, American Spinal Injury Association Impairment Scale; BP, blood pressure; RCT, randomized controlled trial; T, thoracic; L, lumbar; EMG, electromyography; dP/dt, rate of rise of ventricular pressure; PRM, posterior-root muscle.

#### 4.4.1 Invasive neuromodulation: epidural spinal cord stimulation

Epidural spinal cord stimulation (eSCS) involves surgically implanting an electrode array on the dorsal aspect of the spinal cord, typically over lower thoracic or upper lumbar segments, to electrically stimulate spinal circuits. In chronic SCI, pioneering work by Harkema and her colleagues has demonstrated the ability to increase arterial blood pressure to normotensive levels using eSCS in individuals with severe injury-induced hypotension (139). They implanted a 16-electrode epidural array spanning lower lumbar-sacral (L1-S1) spinal segments in four patients and identified stimulation parameters (frequency, pulse width, and electrode configuration) that reproducibly maintained systolic blood pressure around 110 to 120 mmHg during upright positioning. This approach provides a tonic excitatory drive to the sympathetic nervous system below the lesion, restoring vascular tone and adrenergic output, counteracting the loss of supraspinal input (129, 139). Notably, this eSCS can be tuned to avoid significant skeletal muscle activation.

Building on this model, Squair and colleagues systematically mapped the spinal segments that most effectively drive pressor responses and uncovered how eSCS engages sympathetic circuitry (140). In rodent experiments, they showed that continuous stimulation evokes a greater blood pressure increase when targeted to lower thoracic (T11 to T13) “hemodynamic hotspots” with dense areas of sympathetic preganglionic neurons. Further, they provide evidence that these effects depend on recruiting large-diameter posterior root afferents that activate splanchnic sympathetic ganglia via excitatory interneurons, producing α1 adrenergic mediated vasoconstriction. They then encoded the spatial and temporal features into a biomimetic closed-loop controller that titrated eSCS amplitude to a user-defined blood pressure target. This “neuroprosthetic baroreflex” technique restored and stabilized blood pressure within seconds during stimulated orthostatic stress after both acute and chronic SCI in rats. The group has translated this work to a nonhuman primate model with acute complete T3 SCI, showing immediate normalization of blood pressure and maintained stability for extended periods. Finally, the procedure was used in a human with medically refractory orthostatic hypotension due to chronic cervical SCI. Treatment abolished burdensome hemodynamic symptoms allowing for permanent cessation of vasopressor medications.

#### 4.4.2 Limitations and future directions of eSCS

Collectively, these data provide mechanistic and translational support for targeted, feedback-controlled eSCS to replace lost supraspinal autonomic regulation after SCI. Despite the promising results, translation to the acute phase comes with many practical challenges. Stimulator implantation requires neurosurgery in medically unstable trauma patients, which raises risks of infection, surgical stress, and device complications (141, 142). Timing is also an issue. While device implantation during the initial spine surgery could avoid a second operation, effective pressor control has been mapped to lower thoracolumbar segments, so patients with higher level injuries would require a separate distal site procedure (140). In chronic studies, stimulus parameters are individualized and optimized over multiple sessions and postures, which is logistically difficult soon after trauma when patients may be sedated, intubated, or unable to sit safely (131, 139, 140, 143). Cost, regulatory pathways, and the need for multidisciplinary expertise also limit near-term use to specialized centers (144). Even so, the potential to restore hemodynamic stability is driving ongoing clinical trials, such as HemON (Clinicaltrials.gov NCT05111093), which should provide valuable insight and address many of these concerns.

#### 4.4.3 Noninvasive neuromodulation: transcutaneous spinal cord stimulation

Transcutaneous spinal cord stimulation (tSCS) is a noninvasive approach that uses surface electrodes placed on the skin over the spine to deliver electrical currents to dorsal spinal circuits (145). Despite attenuation by soft tissues, tSCS can recruit posterior-root afferents and engage inter- and intrasegmental interneuronal networks that, in turn, increase the excitability of sympathetic preganglionic neurons and enable pressor responses (146, 147). Mechanistically, posterior root recruitment during tSCS appears to activate similar input structures as eSCS, supporting a shared somatoautonomic pathway for vascular control (147).

Early clinical evidence shows that tSCS can normalize blood pressure during orthostatic stress in chronic SCI (148). In a five-participant crossover study of motor complete SCI, monophasic 30 Hz tSCS was delivered at the T7/8 interspinous level (148). This technique restored systolic pressure, improved cardiac contractility, and increased cerebral blood-flow velocity within 60 s of application during head-up tilt, without causing lower-limb muscle contraction. These effects were titrated by current amplitude (10 to 70 mA) to reach a pre-defined normotensive range. This amplitude-dependent increase in blood pressure is consistent with a sympathetically mediated vasoconstrictor mechanism rather than via the skeletal muscle pump.

Subsequent mapping studies helped refine the spatial requirements of tSCS to optimize the hemodynamic effects (149). Engel-Haber and colleagues applied tSCS at various spinal levels in a case series of eight patients with chronic cervical SCI (149). They found that tSCS at lower thoracolumbar levels (T11/12, L1/2, S1/2) yielded a significant increase in blood pressure, whereas cervical and upper thoracic sites produced little or no response. This thoracolumbar “hotspot” pattern complements the epidural mapping work by Squair and colleagues (140).

Two prospective protocols now standardize parameter selection for blood pressure control with tSCS. Peters & colleagues detailed a mapping approach that explores cathode placement at T7/8, T9/10, T11/12, and L1/1, frequencies of 30 and 60 Hz, 1 ms pulse width, with or without a 10 kHz carrier, and amplitudes titrated up to 120 mA while continuously monitoring blood pressure to maintain a seated target of 110 to 120 mmHg in chronic SCI patients (145). In parallel, the randomized sham-controlled MACHINE trial pairs locomotor training with tSCS delivered at T11 and L1 over 12 weeks and includes 24-h ambulatory blood pressure monitoring and head-up tilt testing to quantify orthostatic hypotension and autonomic dysreflexia burden (146). Together, these protocols provide practical spatial and temporal guidelines that make clinical translation more reproducible.

#### 4.4.4 Limitations and future directions of tSCS

A major advantage of tSCS is its rapid deployability and a favorable short-term safety profile. Gel electrodes can be applied to the skin, allowing stimulation to begin within minutes, while parameters can be adjusted in real time to achieve hemodynamic targets (145, 148, 150). Importantly, this intervention can be easily discontinued or repeated as needed without any surgical intervention. However, tSCS also has limitations, particularly during the acute phase of SCI care. A practical challenge is achieving consistent electrode contact and stimulus delivery in immobilized patients (145). Moreover, tSCS is less focal than eSCS and can co-activate sensory and motor fibers, potentially causing off target effects (147, 151). There is evidence of this occurring in chronic SCI patients who received sub-threshold tSCS leading to augmented blood pressure, yet increased hypertensive episodes and precipitated autonomic dysreflexia during noxious stimulation (151). Additionally, the net effects on autonomic regulation remain unclear (151). Several acute and early-subacute tSCS clinical trials aimed at hemodynamic stabilization spanning settings from ICU to inpatient rehabilitation are underway to better identify optimal targets, provide information regarding safety, and highlight long-term effects (NCT06000592; NCT06540859; and NCT07090473).

#### 4.4.5 The future of neuromodulation

Utilizing electrical stimulation in the acute phase could revolutionize how we stabilize patients after SCI. Harnessing these techniques in a step-wise approach using tSCS placed soon after hospitalization and then transitioning to an implanted eSCS later may allow for the earliest intervention. This sequential noninvasive to invasive stimulation approach has been effectively demonstrated in motor recovery literature, but has not yet been employed in cardiovascular function work (152). Mukhametova and colleagues suggest this early approach may allow for the identification of individuals that successfully respond to tSCS, providing justification for transitioning to long-term regulation via implanted eSCS devices (152).

## 5 Discussion

### 5.1 Summary

The current review has explored how acute SCI disrupts autonomic cardiovascular regulation, precipitating hemodynamic instability that amplifies secondary injury and worsens neurological outcomes. SCI-induced loss of descending sympathetic regulation leads to reduced spinal cord perfusion, ischemia–reperfusion injury, endothelial barrier disruption, and intraparenchymal hemorrhage, which collectively exacerbate secondary injury and impede recovery (9–11, 58, 59, 74). In the context of acute SCI, hemodynamic management is not merely supportive care, but is a direct intervention on a process that drives secondary tissue loss and a critical therapeutic target for precision treatment in the hours to days following injury. This underscores the need to refine current practices and advance new strategies to optimize recovery. Although the data synthesized in this review derive predominantly from traumatic SCI cohorts, these findings now shape hemodynamic decisions in non-traumatic and primary vascular cord syndromes where similar autonomic and perfusion disturbances are observed (3, 27–29). The key implication is that trauma-derived hemodynamic targets should serve as a physiologic framework rather than a fixed standard when treating non-traumatic or primary vascular SCI. In practice, blood pressure augmentation and other perfusion-directed strategies should be tailored to the underlying etiology and vascular comorbidities, and applied as trauma-informed benchmarks until prospective data in these populations become available (22, 29).

#### 5.1.1 Reframing MAP targets: timing, thresholds, and limits

Three themes emerged from the MAP literature. First, the earliest intervention offers the most benefit. Independent cohorts show that avoiding even transient hypotensive events in the first 12 to 72 h correlated with better motor recovery, while prehospital hypotension is linked with worse admission grades (11, 14, 88, 93, 94). Second, further bolstering blood pressure augmentation does not necessarily yield greater benefit. Machine learning analyses of intraoperative signals identified an optimal performance window, with the likelihood of neurological improvement decreasing outside this range and evidence of harm when mean values remained above approximately 96 mmHg for prolonged periods (89, 90). Observational ICU data similarly show that strict adherence to a narrow 85 to 90 mmHg target is rarely feasible and often increases pressure variability and exposure to hypertension (21). Third, lower MAP goals may be sufficient for many patients. Two studies found no advantage of maintaining the traditional 85 to 90 mmHg targets compared with more moderate goals, particularly when the priority was to prevent hypotension rather than sustaining prolonged supraphysiologic pressures (96, 97). Collectively, these findings support a pragmatic approach that emphasizes rapid correction of hypotension and maintenance of MAP within a permissive upper range, rather than targeting sustained maximal augmentation. This perspective is reflected in the 2024 multidisciplinary guideline update, which recommends maintaining MAP between a lower bound of 75 to 80 mmHg and an upper bound of 90 to 95 mmHg for 3 to 7 days, while emphasizing the importance of individualized care (22).

#### 5.1.2 Advantages of SCPP-guided therapy over MAP

MAP is a systemic measure that is used as a surrogate for cord perfusion. However, as edema raises intrathecal or intraspinal pressures within a fixed canal, perfusion at the lesion can be compromised despite achieving MAP goals. Managing SCPP directly follows established neurocritical care practices and offers insight at the level of the spinal microenvironment that MAP monitoring alone cannot provide (98, 99). Lumbar CSF drainage is a feasible and generally safe intervention in acute SCI, effectively lowering ITP and increasing SCPP, with time spent above SCPP thresholds shown to predict motor recovery more reliably than targeting MAP alone (15, 101–103). Subdural probes placed at the lesion provide more accurate local pressure measurements than lumbar monitoring, particularly during periods of cord swelling, thereby reducing the risk of underestimation (105). This approach also demonstrates that patient-specific optimal SCPP, derived from the spinal pressure reactivity index, can shift dynamically over time, with deviations from this optimum correlating with metabolic stress and worse outcomes (107, 108). Together, these findings suggest that SCPP-guided care can refine therapeutic windows and reduce reliance on vasopressors by addressing local cord perfusion rather than systemic MAP targets alone (98, 106, 110).

#### 5.1.3 Vasopressor choice and timing: implications for cord perfusion

Current evidence supports NE as the preferred vasopressor in acute SCI because it reliably raises MAP with fewer arrhythmias than DA and provides balanced *α* adrenergic mediated vasoconstriction with modest *β*_1_ inotropy suited for neurogenic shock physiology (111–114, 116). Small clinical and preclinical studies suggest NE may improve SCPP more effectively than DA at comparable MAP levels, but the studies also demonstrate that initiating vasopressors before decompression can exacerbate intraparenchymal hemorrhage (62, 70, 120). These findings emphasize the importance of aligning pharmacological blood pressure management with surgical intervention to minimize risk. PHE is a reasonable alternative when β-adrenergic stimulation is undesirable, although its pure α-adrenergic profile can lower stroke volume and, in preclinical models, has been associated with increased hemorrhage when used prior to decompression (116, 119, 128). Comparative human data between NE and PHE remain limited and confounded, so definitive recommendations await controlled trials (124, 127). Across all vasopressors, the message is consistent: Vasopressors are necessary tools to correct hypotension, but escalation beyond physiologic need carries tradeoffs that may worsen the very secondary injury processes that we aim to prevent (89, 91, 95).

#### 5.1.4 Neuromodulation reframes hemodynamic care from compensation to restoration

Electrical stimulation of somatoautonomic circuits below the lesion can normalize arterial pressure and stabilize cardiovascular control in chronic SCI, often without skeletal muscle co-activation (138, 148). eSCS studies have identified thoracolumbar ‘hemodynamic hotspots’ as critical regions for blood pressure control (140, 147). Closed-loop approaches restored blood pressure with second-to-second precision in preclinical models and were successfully translated to humans with SCI, including cases of refractory hypotension (137–140). tSCS is a noninvasive and rapidly deployable approach that can elevate blood pressure within minutes, though it is less localized and may co-stimulate sensory pathways and precipitate autonomic dysreflexia (145, 147–149, 151). Translating this work to the acute phase requires overcoming several limitations including surgical candidacy for epidural implantation, sedation, immobilization, and infection risk. A staged approach that begins with tSCS in the early phase of injury and transitions to epidural implantation in patients who demonstrate a favorable response could help mitigate these barriers and allow for personalized long-term autonomic rehabilitation (149, 152). This approach mirrors established protocols in motor recovery and warrants investigation for cardiovascular control (130–133).

In considering translation to acute care, it is important to recognize that the first days after SCI are characterized by hemodynamic instability and impaired vascular reflexes. Because neuromodulation exerts direct control over sympathetic outflow and vascular tone, early use could precipitate overshoot hypertension, arrhythmia, hemorrhage, or cerebrovascular complications. Such adverse events have been reported in neurologically intact individuals receiving neuromodulatory devices, whereas systematic safety data in acute SCI are not yet available (141, 142, 151, 153, 154). Early neuromodulation should therefore be regarded as investigational until prospective trials define its risk profile. At the same time, people with SCI have an increased long-term risk of cardiovascular disease and stroke that reflects autonomic dysregulation and cardiometabolic remodeling (155, 156). In chronic SCI, small prospective cohorts indicate that epidural and transcutaneous stimulation can normalize blood pressure and improve indices of cardiac function when delivered with careful monitoring, suggesting a potential to attenuate maladaptive cardiovascular remodeling (137, 139, 142, 148). Neuromodulation therefore warrants further investigation both as a strategy to reduce long-term cardiovascular consequences of SCI and to clarify the safety and efficacy of its use in the acute phase.

#### 5.1.5 From evidence to practice: a framework for hemodynamic management

Taken together, current evidence supports a stepwise, physiology-driven approach that can be implemented now while awaiting further validation from trials of SCPP-guided care and neuromodulation (22, 110). During the early phase of treatment, spanning prehospital care through the first 72 h, priorities include rapid correction of hypoxemia and hypovolemia and prompt avoidance of hypotension. During this period, MAP should be maintained at or above 75 to 80 mmHg while limiting sustained pressures beyond about 95 to 100 mmHg unless individualized data justify higher targets (11, 14, 88). NE is the preferred vasopressor given its efficacy and lower adverse effect profile, but its use should be coordinated with early decompression when indicated (111–114, 116).

Within the first 7 days following SCI, centers with appropriate expertise may utilize SCPP-directed care while in the ICU, utilizing lumbar drain or intraspinal probe placement (15, 102, 103, 108). Lumbar drainage can be used to monitor ITP in addition to allowing CSF drainage to keep SCPP near 60 to 65 mmHg (98, 99, 101). In select post-decompression cases, intraspinal monitoring with subdural probe placement can be considered along with guide expansion duroplasty when compartment physiology is suspected (106, 107, 110). As perfusion stabilizes, vasopressors should be weaned while maintaining the therapeutic MAP window (116).

For persistent hemodynamic lability despite optimized MAP and/or SCPP, adjunct neuromodulation can be trialed with tSCS to stabilize blood pressure (145, 148, 149). Progression to epidural implanted devices can be considered at specialized centers in patients identified as potential responders (131, 133, 138, 140).

### 5.2 Limitations

The current evidence remains limited by small observational studies, heterogeneity in injury characteristics, and potential bias from the most severely injured patients receiving more aggressive treatments, making outcomes difficult to interpret. Direct SCPP monitoring provides valuable insight, but requires invasive techniques and timely expertise, limiting its availability beyond specialized centers. Comparative data on vasopressor choice is lacking, with NE’s advantages over other agents supported mainly by preclinical work and small clinical studies. Neuromodulation has demonstrated promise in chronic SCI, yet its translation to acute care is challenged by surgical candidacy, timing, and the lack of standardized protocols. These gaps reinforce the need for multicenter trials that combine physiologic monitoring with pragmatic outcomes to guide precision hemodynamic management. In addition, this review was intentionally focused on hemodynamic and neuromodulatory strategies for cardiovascular stabilization after acute SCI. Pharmacologic neuroprotective approaches such as riluzole, minocycline, and other antioxidant or anti-inflammatory agents were not reviewed here and have been summarized in detail elsewhere (64, 157, 158). Future work should clarify how best to integrate these pharmacologic strategies with perfusion-targeted and neuromodulatory approaches to address both vascular and cellular mechanisms of secondary injury.

## 6 Conclusion

Acute SCI disrupts descending sympathetic control, leading to hemodynamic instability that exacerbates secondary injury and ultimately worsens neurological recovery. Because of this, hemodynamic management should be treated as a therapeutic intervention, rather than supportive care. Current evidence supports rapid prevention of hypotension, avoidance of excessive hypertension, and strategies that integrate systemic blood pressure management with local cord perfusion. Emerging approaches, including SCPP-guided therapy and staged neuromodulation, offer translational pathways toward personalized precision management. Essential next steps include rigorous multicenter trials to define optimal MAP targets, validation of SCPP-based protocols, and elucidating the role of neuromodulatory interventions for improving neurological and cardiovascular outcomes.

## References

[1] Jain NB Ayers GD Peterson EN Harris MB Morse L O'Connor KC . Traumatic spinal cord injury in the United States, 1993-2012. JAMA. (2015) 313:2236–43. doi: 10.1001/jama.2015.6250, 26057284 PMC4712685

[2] Lasfargues JE Custis D Morrone F Carswell J Nguyen T. A model for estimating spinal cord injury prevalence in the United States. Paraplegia. (1995) 33:62–8. 7753569 10.1038/sc.1995.16

[3] Furlan JC Fehlings MG. Cardiovascular complications after acute spinal cord injury: pathophysiology, diagnosis, and management. Neurosurg Focus. (2008) 25:E13. doi: 10.3171/FOC.2008.25.11.E13, 18980473

[4] Cardile D Calderone A De Luca R Corallo F Quartarone A Calabrò RS. The quality of life in patients with spinal cord injury: assessment and rehabilitation. J Clin Med. (2024) 13:1820. doi: 10.3390/jcm13061820, 38542044 PMC10971730

[5] Burns K Solinsky R. Autonomic impairment is not explained by neurological level of injury or motor-sensory completeness. Spinal Cord. (2024) 62:367–70. doi: 10.1038/s41393-024-00994-7, 38609568 PMC11230852

[6] Draghici A Taylor J. Baroreflex autonomic control in human spinal cord injury: physiology, measurement, and potential alterations. Auton Neurosci. (2018) 209:37–42. doi: 10.1016/j.autneu.2017.08.007, 28844537

[7] Sahota I Ravensbergen H McGrath M Claydon V. Cerebrovascular responses to orthostatic stress after spinal cord injury. J Neurotrauma. (2012) 29:2446–56. doi: 10.1089/neu.2012.2379, 22720841

[8] Teasell RW Arnold JMO Krassioukov A Delaney GA. Cardiovascular consequences of loss of supraspinal control of the sympathetic nervous system after spinal cord injury. Arch Phys Med Rehabil. (2000) 81:506–16. 10768544 10.1053/mr.2000.3848

[9] Eldahan KC Rabchevsky AG. Autonomic dysreflexia after spinal cord injury: systemic pathophysiology and methods of management. Auton Neurosci. (2018) 209:59–70. doi: 10.1016/j.autneu.2017.05.002, 28506502 PMC5677594

[10] Tator CH Fehlings MG. Review of the secondary injury theory of acute spinal cord trauma with emphasis on vascular mechanisms. J Neurosurg. (1991) 75:15–26. 2045903 10.3171/jns.1991.75.1.0015

[11] Hawryluk G Whetstone W Saigal R Ferguson A Talbott J Bresnahan J . Mean arterial blood pressure correlates with neurological recovery after human spinal cord injury: analysis of high-frequency physiologic data. J Neurotrauma. (2015) 32:1958–67. doi: 10.1089/neu.2014.3778, 25669633 PMC4677564

[12] Lee YS Kim KT Kwon BK. Hemodynamic Management of Acute Spinal Cord Injury: a literature review. Neurospine. (2021) 18:7–14. doi: 10.14245/ns.2040144.072, 33211951 PMC8021842

[13] Picetti E Iaccarino C Coimbra R Abu-Zidan F Tebala GD Balogh ZJ . The acute phase management of spinal cord injury affecting polytrauma patients: the ASAP study. World J Emerg Surg. (2022) 17:20. doi: 10.1186/s13017-022-00422-2, 35468806 PMC9036814

[14] Haldrup M Dyrskog S Thygesen MM Kirkegaard H Kasch H Rasmussen MM. Initial blood pressure is important for long-term outcome after traumatic spinal cord injury. J Neurosurg Spine. (2020) 33:256–60. doi: 10.3171/2020.1.SPINE191005, 32197239

[15] Yue JK Hemmerle DD Winkler EA Thomas LH Fernandez XD Kyritsis N . Clinical implementation of novel spinal cord perfusion pressure protocol in acute traumatic spinal cord injury at U.S. level I trauma center: TRACK-SCI study. World Neurosurg. (2020) 133:e391–6. doi: 10.1016/j.wneu.2019.09.044, 31526882

[16] Anderson D Nicolosi G Means E Hartley L. Effects of laminectomy on spinal cord blood flow. J Neurosurg. (1978) 48:232–8. 624972 10.3171/jns.1978.48.2.0232

[17] Wallace C Tator C. Successful improvement of blood pressure, cardiac output, and spinal cord blood flow after experimental spinal cord injury. Neurosurgery. (1987) 20:710–5. 2439939 10.1227/00006123-198705000-00006

[18] Menacho ST Floyd C. Current practices and goals for mean arterial pressure and spinal cord perfusion pressure in acute traumatic spinal cord injury: defining the gaps in knowledge. J Spinal Cord Med. (2021) 44:350–6. doi: 10.1080/10790268.2019.1660840, 31525138 PMC8081322

[19] Gee CM Kwon BK. Significance of spinal cord perfusion pressure following spinal cord injury: a systematic scoping review. J Clinical Orthopaedics Trauma. (2022) 34:102024. doi: 10.1016/j.jcot.2022.102024, 36147378 PMC9486559

[20] Kong CY Hosseini AM Belanger LM Ronco JJ Paquette SJ Boyd MC . A prospective evaluation of hemodynamic management in acute spinal cord injury patients. Spinal Cord. (2013) 51:466–71. doi: 10.1038/sc.2013.32, 23743499

[21] Gee CM Tsang A Bélanger LM Ritchie L Ailon T Paquette S . All over the MAP: describing pressure variability in acute spinal cord injury. Spinal Cord. (2022) 60:470–5. doi: 10.1038/s41393-022-00802-0, 35418625

[22] Kwon BK Tetreault LA Martin AR Arnold PM Marco RAW Newcombe VFJ . A clinical practice guideline for the management of patients with acute spinal cord injury: recommendations on hemodynamic management. Glob Spine J. (2024) 14:187S–211S. doi: 10.1177/21925682231202348, 38526923 PMC10964888

[23] Ahuja CS Nori S Tetreault L Wilson J Kwon B Harrop J . Traumatic spinal cord injury-repair and regeneration. Neurosurgery. (2017) 80:S9–s22. doi: 10.1093/neuros/nyw080, 28350947

[24] Kwon BK Tetzlaff W Grauer JN Beiner J Vaccaro AR. Pathophysiology and pharmacologic treatment of acute spinal cord injury. Spine J. (2004) 4:451–64. doi: 10.1016/j.spinee.2003.07.007, 15246307

[25] Fehlings MG Tetreault LA Hachem L Evaniew N Ganau M McKenna SL . An update of a clinical practice guideline for the management of patients with acute spinal cord injury: recommendations on the role and timing of decompressive surgery. Glob Spine J. (2024) 14:174S–86S. doi: 10.1177/21925682231181883, 38526922 PMC10964895

[26] Hubertus V Badhiwala JH Hejrati N Nouri A Ter Wengel PV Farahbakhsh F . AO spine clinical practice recommendations for the surgical management of acute traumatic spinal cord injury: contemporary concepts. Glob Spine J. (2025) 15:3572–9. doi: 10.1177/21925682251350941 PMC12146309 40483581

[27] Krassioukov A Claydon VE. The clinical problems in cardiovascular control following spinal cord injury: an overview. Prog Brain Res. (2006) 152:223–9. doi: 10.1016/S0079-6123(05)52014-4, 16198703

[28] Partida E Mironets E Hou S Tom VJ. Cardiovascular dysfunction following spinal cord injury. Neural Regen Res. (2016) 11:189–94. doi: 10.4103/1673-5374.177707, 27073353 PMC4810964

[29] Naik A Houser SL Moawad CM Iyer RK Arnold PM. Noniatrogenic spinal cord ischemia: a patient level meta-analysis of 125 case reports and series. Surg Neurol Int. (2022) 13:228. doi: 10.25259/SNI_1252_2021, 35855116 PMC9282799

[30] Gordan R Gwathmey JK Xie LH. Autonomic and endocrine control of cardiovascular function. World J Cardiol. (2015) 7:204–14. doi: 10.4330/wjc.v7.i4.204, 25914789 PMC4404375

[31] Ziegler KA Engelhardt S Carnevale D Mcalpine CS Guzik TJ Dimmeler S . Neural mechanisms in cardiovascular health and disease. Circ Res. (2025) 136:1233–61. doi: 10.1161/CIRCRESAHA.125.325580, 40403111

[32] Purves D Augustine G Fitzpatrick D Hall W LaMantia A-S. Neuroscience. 6th ed. Oxford, United Kingdom: Oxford University Press (2018).

[33] Salman IM. Major autonomic neuroregulatory pathways underlying short- and long-term control of cardiovascular function. Curr Hypertens Rep. (2016) 18:52. doi: 10.1007/s11906-016-0625-x, 26838031

[34] Brunton L Hilal-Dandan R Knollmann B. Goodman & Gilman’s the pharmacological basis of therapeutics. 13th ed. New York, NY: McGraw-Hill Education (2018).

[35] Hall J. Guyton and Hall textbook of medical physiology. 14th ed. Philadelphia, PA: Elsevier (2021).

[36] Camargo E Samuels M. Cardiac and autonomic manifestations of stroke In: Stroke syndromes. 3rd ed, eds. Caplan LR van Gijn J. Cambridge: Cambridge University Press (2012). 294–305.

[37] Dampney RA. Functional organization of central pathways regulating the cardiovascular system. Physiol Rev. (1994) 74:323–64. 8171117 10.1152/physrev.1994.74.2.323

[38] Guyenet PG. The sympathetic control of blood pressure. Nat Rev Neurosci. (2006) 7:335–46. doi: 10.1038/nrn1902, 16760914

[39] Guyenet PG Stornetta RL. Rostral ventrolateral medulla, retropontine region and autonomic regulations. Auton Neurosci. (2022) 237:102922. doi: 10.1016/j.autneu.2021.102922, 34814098

[40] Ruffinazzi M Dusi V. Central nervous system Management of Autonomic Cardiovascular Control. Brain Heart Dynamics, eds. Govoni S Politi P Vanoli E. Cham: Springer. (2021) p. 1–27. doi: 10.1007/978-3-319-90305-7_65-1

[41] Westfall DP Westfall TC. Neurotransmission: the autonomic and somatic motor nervous systems In: Brunton LL Chabner BA Knollmann BC, editors. Goodman & Gilman's the pharmacological basis of therapeutics. US: McGraw-Hill (2011). 215–58.

[42] Loewy A Spyer K In: Loewy AD Spyer KM, editors. Central regulation of autonomic functions. UK: Oxford University Press (1990)

[43] Nakamura K Morrison SF. Central sympathetic network for thermoregulatory responses to psychological stress. Auton Neurosci. (2022) 237:102918. doi: 10.1016/j.autneu.2021.102918, 34823147

[44] Savić B Murphy D Japundžić-Žigon N. The paraventricular nucleus of the hypothalamus in control of blood pressure and blood pressure variability. Front Physiol. (2022) 13:941. doi: 10.3389/fphys.2022.858941, 35370790 PMC8966844

[45] Sladek CD Michelini LC Stachenfeld NS Stern JE Urban JH. Endocrine-autonomic linkages. Compr Physiol. (2015) 5:1281–323. doi: 10.1002/j.2040-4603.2015.tb00634.x, 26140719

[46] Antunes-Rodrigues J Ruginsk SG Mecawi AS Margatho LO Cruz JC Vilhena-Franco T . Mapping and signaling of neural pathways involved in the regulation of hydromineral homeostasis. Braz J Med Biol Res. (2013) 46:327–38. doi: 10.1590/1414-431X20132788, 23579631 PMC3854407

[47] Benarroch EE. Periaqueductal gray. Neurology. (2012) 78:210–7. doi: 10.1212/WNL.0b013e31823fcdee, 22249496

[48] Simmons WK Avery JA Barcalow JC Bodurka J Drevets WC Bellgowan P. Keeping the body in mind: insula functional organization and functional connectivity integrate interoceptive, exteroceptive, and emotional awareness. Hum Brain Mapp. (2013) 34:2944–58. doi: 10.1002/hbm.22113, 22696421 PMC6870113

[49] Rossi GM Regolisti G Peyronel F Fiaccadori E. Recent insights into sodium and potassium handling by the aldosterone-sensitive distal nephron: a review of the relevant physiology. J Nephrol. (2020) 33:431–45. doi: 10.1007/s40620-019-00684-1, 31950375

[50] Ranjan AK Gulati A. Controls of central and peripheral blood pressure and hemorrhagic/hypovolemic shock. J Clin Med. (2023) 12:1108. doi: 10.3390/jcm12031108, 36769755 PMC9917827

[51] Fisher JP Ogoh S Young CN Keller DM Fadel PJ. Exercise intensity influences cardiac baroreflex function at the onset of isometric exercise in humans. J Appl Physiol. (2007) 103:941–7. doi: 10.1152/japplphysiol.00412.2007, 17585044

[52] Fisher JP Young CN Fadel PJ. Autonomic adjustments to exercise in humans. Compr Physiol. (2015) 5:614. doi: 10.1002/j.2040-4603.2015.tb00614.x, 25880502

[53] Brignole M Moya A De Lange FJ Deharo J-C Elliott PM Fanciulli A . 2018 ESC guidelines for the diagnosis and management of syncope. Eur Heart J. (2018) 39:1883–948. doi: 10.1093/eurheartj/ehy037, 29562304

[54] Mosqueda-Garcia R Furlan R Md JT Fernandez-Violante R. The elusive pathophysiology of Neurally mediated Syncope. Circulation. (2000) 102:2898–906. doi: 10.1161/01.CIR.102.23.2898, 11104751

[55] Wulf MJ Tom VJ. Consequences of spinal cord injury on the sympathetic nervous system. Front Cell Neurosci. (2023) 17:253. doi: 10.3389/fncel.2023.999253, 36925966 PMC10011113

[56] Claydon VE Steeves JD Krassioukov A. Orthostatic hypotension following spinal cord injury: understanding clinical pathophysiology. Spinal Cord. (2006) 44:341–51. doi: 10.1038/sj.sc.3101855, 16304564

[57] Massetti J Stein D. Spinal cord injury In: Neurocritical care for the advanced practice clinician, eds. White JL Sheth KN. Cham: Springer (2018). 269–88.

[58] Khaing ZZ Cates LN DeWees DM Hannah A Mourad P Bruce M . Contrast-enhanced ultrasound to visualize hemodynamic changes after rodent spinal cord injury. J Neurosurg Spine. (2018) 29:306–13. doi: 10.3171/2018.1.SPINE171202, 29905521

[59] Mautes AE Weinzierl MR Donovan F Noble LJ. Vascular events after spinal cord injury: contribution to secondary pathogenesis. Phys Ther. (2000) 80:673–87. doi: 10.1093/ptj/80.7.673, 10869130

[60] Zhou R Li J Chen Z Wang R Shen Y Zhang R . Pathological hemodynamic changes and leukocyte transmigration disrupt the blood–spinal cord barrier after spinal cord injury. J Neuroinflammation. (2023) 20:787. doi: 10.1186/s12974-023-02787-w, 37210532 PMC10200062

[61] Fehlings MG Tator CH Linden RD. The relationships among the severity of spinal cord injury, motor and somatosensory evoked potentials and spinal cord blood flow. Electroencephalography Clin Neurophysiol/Evoked Potentials Section. (1989) 74:241–59. 10.1016/0168-5597(89)90055-5 2471626

[62] Soubeyrand M Dubory A Laemmel E Court C Vicaut E Duranteau J. Effect of norepinephrine on spinal cord blood flow and parenchymal hemorrhage size in acute-phase experimental spinal cord injury. Eur Spine J. (2014) 23:658–65. doi: 10.1007/s00586-013-3086-9, 24232597 PMC3940804

[63] Sharif H Hou S. Autonomic dysreflexia: a cardiovascular disorder following spinal cord injury. Neural Regen Res. (2017) 12:1390–400. doi: 10.4103/1673-5374.215241, 29089975 PMC5649450

[64] Liu NK Xu XM. Neuroprotection and its molecular mechanism following spinal cord injury. Neural Regen Res. (2012) 7:2051–62. doi: 10.3969/j.issn.1673-5374.2012.26.007, 25624837 PMC4296426

[65] Kumar H Ropper AE Lee S-H Han I. Propitious therapeutic modulators to prevent blood-spinal cord barrier disruption in spinal cord injury. Mol Neurobiol. (2017) 54:3578–90. doi: 10.1007/s12035-016-9910-6, 27194298

[66] Monteiro S Pinho AG Macieira M Serre-Miranda C Cibrão JR Lima R . Splenic sympathetic signaling contributes to acute neutrophil infiltration of the injured spinal cord. J Neuroinflammation. (2020) 17:282. doi: 10.1186/s12974-020-01945-8, 32967684 PMC7513542

[67] Bhatt M Sharma M Das B. The role of inflammatory cascade and reactive astrogliosis in glial scar formation post-spinal cord injury. Cell Mol Neurobiol. (2024) 44:519. doi: 10.1007/s10571-024-01519-9, 39579235 PMC11585509

[68] Prüss H Tedeschi A Thiriot A Lynch L Loughhead SM Stutte S . Spinal cord injury-induced immunodeficiency is mediated by a sympathetic-neuroendocrine adrenal reflex. Nat Neurosci. (2017) 20:1549–59. doi: 10.1038/nn.4643, 28920935

[69] Noble BT Brennan FH Popovich PG. The spleen as a neuroimmune interface after spinal cord injury. J Neuroimmunol. (2018) 321:1–11. doi: 10.1016/j.jneuroim.2018.05.007, 29957379

[70] Cheung A Streijger F So K Okon EB Manouchehri N Shortt K . Relationship between early vasopressor administration and spinal cord hemorrhage in a porcine model of acute traumatic spinal cord injury. J Neurotrauma. (2020) 37:1696–707. doi: 10.1089/neu.2019.6781, 32233727

[71] Stokum JA Cannarsa GJ Wessell AP Shea P Wenger N Simard JM. When the blood hits your brain: the neurotoxicity of Extravasated blood. Int J Mol Sci. (2021) 22:5132. doi: 10.3390/ijms22105132, 34066240 PMC8151992

[72] Rathore KI Kerr BJ Redensek A López-Vales R Jeong SY Ponka P . Ceruloplasmin protects injured spinal cord from Iron-mediated oxidative damage. J Neurosci. (2008) 28:12736–47. doi: 10.1523/JNEUROSCI.3649-08.2008, 19036966 PMC6671786

[73] Yoshizaki S Kijima K Hara M Saito T Tamaru T Tanaka M . Tranexamic acid reduces heme cytotoxicity via the TLR4/TNF axis and ameliorates functional recovery after spinal cord injury. J Neuroinflammation. (2019) 16:160. doi: 10.1186/s12974-019-1536-y, 31358003 PMC6661785

[74] Strain MM Johnston DT Baine RE Reynolds JA Huang YJ Henwood MK . Hemorrhage and locomotor deficits induced by pain input after spinal cord injury are partially mediated by changes in hemodynamics. J Neurotrauma. (2021) 38:3406–30. doi: 10.1089/neu.2021.0219, 34652956 PMC8713547

[75] Grau JW Huang YJ Turtle JD Strain MM Miranda RC Garraway SM . When pain hurts: nociceptive stimulation induces a state of maladaptive plasticity and impairs recovery after spinal cord injury. J Neurotrauma. (2017) 34:1873–90. doi: 10.1089/neu.2016.4626, 27788626 PMC5444485

[76] Grau JW Washburn SN Hook MA Ferguson AR Crown ED Garcia G . Uncontrollable stimulation undermines recovery after spinal cord injury. J Neurotrauma. (2004) 21:1795–817. doi: 10.1089/neu.2004.21.1795, 15684770

[77] Flanders AE Spettell CM Tartaglino LM Friedman DP Herbison GJ. Forecasting motor recovery after cervical spinal cord injury: value of MR imaging. Radiology. (1996) 201:649–55. 8939210 10.1148/radiology.201.3.8939210

[78] Konomi T Suda K Ozaki M Harmon SM Komatsu M Iimoto S . Predictive factors for irreversible motor paralysis following cervical spinal cord injury. Spinal Cord. (2021) 59:554–62. doi: 10.1038/s41393-020-0513-8, 32632174

[79] Ryken TC Hurlbert RJ Hadley MN Aarabi B Dhall SS Gelb DE . The acute cardiopulmonary Management of Patients with Cervical Spinal Cord Injuries. Neurosurgery. (2013) 72:84–92. doi: 10.1227/NEU.0b013e318276ee16, 23417181

[80] Fehlings MG Vaccaro A Wilson JR Singh A Harrop JS Aarabi B . Early versus delayed decompression for traumatic cervical spinal cord injury: results of the surgical timing in acute spinal cord injury study (STASCIS). PLoS One. (2012) 7:e32037. doi: 10.1371/journal.pone.0032037, 22384132 PMC3285644

[81] Saadeh YS Smith BW Joseph JR Jaffer SY Buckingham MJ Oppenlander ME . The impact of blood pressure management after spinal cord injury: a systematic review of the literature. Neurosurg Focus. (2017) 43:E20. doi: 10.3171/2017.8.FOCUS17428, 29088944

[82] Raper R. Delayed, transient quadriplegia; the importance of spinal cord perfusion. BMJ Case Rep. (2022) 15:501. doi: 10.1136/bcr-2021-246501, 35523515 PMC9083389

[83] Medicine CfSC. Early acute management in adults with spinal cord injury: A clinical practice guideline for health-care professionals. Washington, DC: Paralyzed Veterans of America (2008). 10.1043/1079-0268-31.4.408 PMC2582434 18959359

[84] Consortium for Spinal Cord Medicine. Early acute management in adults with spinal cord injury: a clinical practice guideline for health-care professionals. J Spinal Cord Med. (2008) 31:403–79. doi: 10.1043/1079-0268-31.4.408 18959359 PMC2582434

[85] Levi L Wolf A Belzberg H. Hemodynamic parameters in patients with acute cervical cord trauma: description, intervention, and prediction of outcome. Neurosurgery. (1993) 33:1007–17. 8133985

[86] Vale FL Burns J Jackson AB Hadley MN. Combined medical and surgical treatment after acute spinal cord injury: results of a prospective pilot study to assess the merits of aggressive medical resuscitation and blood pressure management. J Neurosurg. (1997) 87:239–46. 9254087 10.3171/jns.1997.87.2.0239

[87] Walters BC Hadley MN Hurlbert RJ Aarabi B Dhall SS Gelb DE . Guidelines for the management of acute cervical spine and spinal cord injuries: 2013 update. Neurosurgery. (2013) 60:82–91. doi: 10.1227/01.neu.0000430319.32247.7f, 23839357

[88] Clark JM Bednarz JM Batchelor PE Skeers P Freeman BJC. Prehospital cardiovascular autoregulatory disturbances correlate with the functional neuroanatomy of acute spinal cord injury. Spine. (2023) 48:428–35. doi: 10.1097/BRS.0000000000004571, 36577080

[89] Torres-Espín A Haefeli J Ehsanian R Torres D Almeida CA Huie JR . Topological network analysis of patient similarity for precision management of acute blood pressure in spinal cord injury. eLife. (2021) 10:8015. doi: 10.7554/eLife.68015, 34783309 PMC8639149

[90] Chou A Torres-Espin A Kyritsis N Huie JR Khatry S Funk J . Expert-augmented automated machine learning optimizes hemodynamic predictors of spinal cord injury outcome. PLoS One. (2022) 17:254. doi: 10.1371/journal.pone.0265254, 35390006 PMC8989303

[91] Almeida CA Torres-Espin A Huie JR Sun D Noble-Haeusslein LJ Young W . Excavating FAIR data: the case of the multicenter animal spinal cord injury study (MASCIS), blood pressure, and neuro-recovery. Neuroinformatics. (2021) 20:39. doi: 10.1007/s12021-021-09512-z 33651310 PMC9015816

[92] Lariccia AK Sperwer K Lieber ML Spalding MC. Mean arterial pressure (MAP) augmentation in traumatic spinal cord injuries: early hyperperfusion treatment influences neurologic outcomes. J Spinal Cord Med. (2024) 47:918–25. doi: 10.1080/10790268.2023.2223447, 37428455 PMC11533261

[93] Weinberg JA Farber SH Kalamchi LD Brigeman ST Bohl MA Varda BM . Mean arterial pressure maintenance following spinal cord injury: does meeting the target matter? J Trauma Acute Care Surg. (2021) 90:97–106. doi: 10.1097/TA.0000000000002953, 33003016

[94] Catapano JS John Hawryluk GW Whetstone W Saigal R Ferguson A Talbott J . Higher mean arterial pressure values correlate with neurologic improvement in patients with initially complete spinal cord injuries. World Neurosurg. (2016) 96:72–9. doi: 10.1016/j.wneu.2016.08.053 27565460 PMC5127746

[95] Drotleff N Jansen O Weckwerth C Aach M Schildhauer TA Waydhas C . Pilot study: advanced haemodynamic monitoring after acute spinal cord injury-keep the pressure up? BMC Anesthesiol. (2022) 22:277. doi: 10.1186/s12871-022-01806-2, 36050640 PMC9434085

[96] Långsjö J Jordan S Laurila S Paaso M Thesleff T Huhtala H . Traumatic cervical spinal cord injury: comparison of two different blood pressure targets on neurological recovery. Acta Anaesthesiol Scand. (2024) 68:493–501. doi: 10.1111/aas.14372, 38228292

[97] Martin ND Kepler C Zubair M Sayadipour A Cohen M Weinstein M. Increased mean arterial pressure goals after spinal cord injury and functional outcome. J Emerg Trauma Shock. (2015) 8:94–8. doi: 10.4103/0974-2700.155507, 25949039 PMC4411584

[98] Werndle MC Saadoun S Phang I Czosnyka M Varsos GV Czosnyka ZH . Monitoring of spinal cord perfusion pressure in acute spinal cord injury: initial findings of the injured spinal cord pressure evaluation study*. Crit Care Med. (2014) 42:646–55. doi: 10.1097/CCM.0000000000000028, 24231762

[99] Saadoun S Papadopoulos MC. Targeted perfusion therapy in spinal cord trauma. Neurotherapeutics. (2020) 17:511–21. doi: 10.1007/s13311-019-00820-6, 31916236 PMC7283409

[100] Evaniew N Davies B Farahbakhsh F Fehlings MG Ganau M Graves D . Interventions to optimize spinal cord perfusion in patients with acute traumatic spinal cord injury: an updated systematic review. Glob Spine J. (2024) 14:58s–79s. doi: 10.1177/21925682231218737 PMC10964891 38526931

[101] Kwon BK Curt A Belanger LM Bernardo A Chan D Markez JA . Intrathecal pressure monitoring and cerebrospinal fluid drainage in acute spinal cord injury: a prospective randomized trial. J Neurosurg Spine. (2009) 10:181–93. doi: 10.3171/2008.10.SPINE08217, 19320576

[102] Squair JW Bélanger LM Tsang A Ritchie L Mac-Thiong JM Parent S . Spinal cord perfusion pressure predicts neurologic recovery in acute spinal cord injury. Neurology. (2017) 89:1660–7. doi: 10.1212/WNL.0000000000004519, 28916535

[103] Squair JW Bélanger LM Tsang A Ritchie L Mac-Thiong J-M Parent S . Empirical targets for acute hemodynamic management of individuals with spinal cord injury. Neurology. (2019) 93:e1205–11. doi: 10.1212/WNL.0000000000008125 31409736

[104] Streijger F So K Manouchehri N Tigchelaar S Lee JHT Okon EB . Changes in pressure, hemodynamics, and metabolism within the spinal cord during the first 7 days after injury using a porcine model. J Neurotrauma. (2017) 34:3336–50. doi: 10.1089/neu.2017.5034, 28844181

[105] Hogg FRA Gallagher MJ Kearney S Zoumprouli A Papadopoulos MC Saadoun S. Acute spinal cord injury: monitoring lumbar cerebrospinal fluid provides limited information about the injury site. J Neurotrauma. (2020) 37:1156–64. doi: 10.1089/neu.2019.6789, 32024422

[106] Phang I Werndle MC Saadoun S Varsos G Czosnyka M Zoumprouli A . Expansion Duroplasty improves Intraspinal pressure, spinal cord perfusion pressure, and vascular pressure reactivity index in patients with traumatic spinal cord injury: injured spinal cord pressure evaluation study. J Neurotrauma. (2015) 32:865–74. doi: 10.1089/neu.2014.3668, 25705999 PMC4492612

[107] Varsos GV Werndle MC Czosnyka ZH Smielewski P Kolias AG Phang I . Intraspinal pressure and spinal cord perfusion pressure after spinal cord injury: an observational study. J Neurosurg Spine. (2015) 23:763–71. doi: 10.3171/2015.3.SPINE14870, 26273764

[108] Chen S Smielewski P Czosnyka M Papadopoulos MC Saadoun S. Continuous monitoring and visualization of optimum spinal cord perfusion pressure in patients with acute cord injury. J Neurotrauma. (2017) 34:2941–9. doi: 10.1089/neu.2017.4982, 28351230

[109] Saadoun S Asif H Papadopoulos MC. The concepts of intra spinal pressure (ISP), intra thecal pressure (ITP), and spinal cord perfusion pressure (SCPP) in acute, severe traumatic spinal cord injury: narrative review. Brain Spine. (2024) 4:103919. doi: 10.1016/j.bas.2024.103919, 39654909 PMC11626061

[110] Ruiz-Cardozo MA Barot K Yahanda AT Singh SP Trevino G Yakdan S . Invasive devices to monitor the intraspinal perfusion pressure in the hemodynamic management of acute spinal cord injury: a systematic scoping review. Acta Neurochir. (2024) 166:283. doi: 10.1007/s00701-024-06283-9, 39382579

[111] Taman M Abdulrazeq H Chuck C Sastry RA Ali R Chen CC . Vasopressor use in acute spinal cord injury: current evidence and clinical implications. J Clin Med. (2025) 14:902. doi: 10.3390/jcm14030902, 39941573 PMC11818478

[112] Inoue T Manley GT Patel N Whetstone WD. Medical and surgical management after spinal cord injury: vasopressor usage, early surgerys, and complications. J Neurotrauma. (2014) 31:284–91. doi: 10.1089/neu.2013.3061, 24020382

[113] De Backer D Aldecoa C Njimi H Vincent JL. Dopamine versus norepinephrine in the treatment of septic shock: a meta-analysis*. Crit Care Med. (2012) 40:725–30. doi: 10.1097/CCM.0b013e31823778ee, 22036860

[114] De Backer D Biston P Devriendt J Madl C Chochrad D Aldecoa C . Comparison of dopamine and norepinephrine in the treatment of shock. N Engl J Med. (2010) 362:779–89. doi: 10.1056/NEJMoa0907118, 20200382

[115] Westfall TC Macarthur H Westfall DP. Adrenergic agonists and antagonists In: Brunton LL Hilal-Dandan R Knollmann BC, editors. Goodman & Gilman's: The pharmacological basis of therapeutics, 13e. New York, NY: McGraw-Hill Education (2017)

[116] Russell JA. Vasopressor therapy in critically ill patients with shock. Intensive Care Med. (2019) 45:1503–17. doi: 10.1007/s00134-019-05801-z, 31646370

[117] Magda S Margulescu AD. Comparison of dopamine and norepinephrine in the treatment of shock. Maedica (Bucur). (2010) 5:69–70. 21977124 PMC3150088

[118] Patel GP Grahe JS Sperry M Singla S Elpern E Lateef O . Efficacy and safety of dopamine versus norepinephrine in the management of septic shock. Shock. (2010) 33:375–80. doi: 10.1097/SHK.0b013e3181c6ba6f, 19851126

[119] Streijger F So K Manouchehri N Gheorghe A Okon EB Chan RM . A direct comparison between norepinephrine and phenylephrine for augmenting spinal cord perfusion in a porcine model of spinal cord injury. J Neurotrauma. (2018) 35:1345–57. doi: 10.1089/neu.2017.5285, 29338544

[120] Altaf F Griesdale DE Belanger L. The differential effects of norepinephrine and dopamine on cerebrospinal fluid pressure and spinal cord perfusion pressure after acute human spinal cord injury. Spinal Cord. (2017) 55:33–8. doi: 10.1038/sc.2016.79, 27271117

[121] Livesey M Jauregui JJ Hamaker MC Pensy RA Langhammer CG Eglseder WA. Management of vasopressor induced ischemia. J Orthop. (2020) 22:497–502. doi: 10.1016/j.jor.2020.10.012, 33100742 PMC7577194

[122] Bangash MN Kong ML Pearse RM. Use of inotropes and vasopressor agents in critically ill patients. Br J Pharmacol. (2012) 165:2015–33. doi: 10.1111/j.1476-5381.2011.01588.x, 21740415 PMC3413841

[123] Meier-Hellmann A Bredle DL Specht M Spies C Hannemann L Reinhart K. The effects of low-dose dopamine on splanchnic blood flow and oxygen uptake in patients with septic shock. Intensive Care Med. (1997) 23:31–7. 9037637 10.1007/s001340050287

[124] Readdy WJ Whetstone WD Ferguson AR Talbott JF Inoue T Saigal R . Complications and outcomes of vasopressor usage in acute traumatic central cord syndrome. J Neurosurg Spine. (2015) 23:574–80. doi: 10.3171/2015.2.SPINE14746, 26230417

[125] Agarwal N Blitstein J Lui A Torres-Espin A Vasnarungruengkul C Burke J . Hypotension requiring vasopressor treatment and increased cardiac complications in elderly spinal cord injury patients: a prospective TRACK-SCI registry study. J Neurosurg Spine. (2023) 38:735–43. doi: 10.3171/2023.2.SPINE221043, 36933260

[126] Readdy WJ Saigal R Whetstone WD Mefford AN Ferguson AR Talbott JF . Failure of mean arterial pressure goals to improve outcomes following penetrating spinal cord injury. Neurosurgery. (2016) 79:708–14. doi: 10.1227/NEU.0000000000001249, 27759678

[127] Hashemaghaie M Ohnuma T Sajdeya R Khandelwal S Yanez ND Krishnamoorthy V . Early vasopressor utilization in critically ill patients with acute traumatic spinal cord injury: a retrospective cohort study. Crit Care Med. (2025) 53:e1952–62. doi: 10.1097/CCM.0000000000006791, 40693862 PMC12617987

[128] Ko C-C Lee P-H Lee J-S Lee K-Z. Spinal decompression surgery may alleviate vasopressor-induced spinal hemorrhage and extravasation during acute cervical spinal cord injury in rats. Spine J. (2024) 24:519–33. doi: 10.1016/j.spinee.2023.09.021, 37793474

[129] Burns M Solinsky R. Toward rebalancing blood pressure instability after spinal cord injury with spinal cord electrical stimulation: a mini review and critique of the evolving literature. Auton Neurosci. (2022) 237:102905. doi: 10.1016/j.autneu.2021.102905, 34800845 PMC9280330

[130] Courtine G Song B Roy RR Zhong H Herrmann JE Ao Y . Recovery of supraspinal control of stepping via indirect propriospinal relay connections after spinal cord injury. Nat Med. (2008) 14:69–74. doi: 10.1038/nm1682, 18157143 PMC2916740

[131] Angeli C Rejc E Boakye M Herrity A Mesbah S Hubscher C . Targeted selection of stimulation parameters for restoration of motor and autonomic function in individuals with spinal cord injury. Neuromodulation. (2024) 27:645–60. doi: 10.1016/j.neurom.2023.03.014, 37140522 PMC10624649

[132] Gill ML Grahn PJ Calvert JS Linde MB Lavrov IA Strommen JA . Neuromodulation of lumbosacral spinal networks enables independent stepping after complete paraplegia. Nat Med. (2018) 24:1677–82. doi: 10.1038/s41591-018-0175-7, 30250140

[133] Wagner FB Mignardot J-B Le Goff-Mignardot CG Demesmaeker R Komi S Capogrosso M . Targeted neurotechnology restores walking in humans with spinal cord injury. Nature. (2018) 563:7729. doi: 10.1038/s41586-018-0649-2 30382197

[134] Harkema S Gerasimenko Y Hodes J Burdick J Angeli C Chen Y . Effect of epidural stimulation of the lumbosacral spinal cord on voluntary movement, standing, and assisted stepping after motor complete paraplegia: a case study. Lancet. (2011) 377:1938–47. doi: 10.1016/S0140-6736(11)60547-3, 21601270 PMC3154251

[135] Edgerton VR Harkema S. Epidural stimulation of the spinal cord in spinal cord injury: current status and future challenges. Expert Rev Neurother. (2011) 11:1351–3. doi: 10.1586/ern.11.129, 21955190 PMC3361963

[136] Rejc E Angeli C Harkema S. Effects of lumbosacral spinal cord epidural stimulation for standing after chronic complete paralysis in humans. PLoS One. (2015) 10:e0133998. doi: 10.1371/journal.pone.0133998, 26207623 PMC4514797

[137] West CR Phillips AA Squair JW Williams AM Walter M Lam T . Association of Epidural Stimulation with Cardiovascular Function in an individual with spinal cord injury. JAMA Neurol. (2018) 75:630–2. doi: 10.1001/jamaneurol.2017.5055, 29459943 PMC5885254

[138] Harkema SJ Legg Ditterline B Wang S Aslan S Angeli CA Ovechkin A . Epidural spinal cord stimulation training and sustained recovery of cardiovascular function in individuals with chronic cervical spinal cord injury. JAMA Neurol. (2018) 75:1569–71. doi: 10.1001/jamaneurol.2018.2617, 30242310 PMC6583212

[139] Harkema SJ Wang S Angeli CA Chen Y Boakye M Ugiliweneza B . Normalization of blood pressure with spinal cord epidural stimulation after severe spinal cord injury. Front Hum Neurosci. (2018) 12:83. doi: 10.3389/fnhum.2018.00083, 29568266 PMC5852107

[140] Squair JW Gautier M Mahe L Soriano JE Rowald A Bichat A . Neuroprosthetic baroreflex controls haemodynamics after spinal cord injury. Nature. (2021) 590:308–14. doi: 10.1038/s41586-020-03180-w, 33505019

[141] Hoelzer BC Bendel MA Deer TR Eldrige JS Walega DR Wang Z . Spinal cord stimulator implant infection rates and risk factors: a multicenter retrospective study. Neuromodulation. (2017) 20:558–62. doi: 10.1111/ner.12609, 28493599

[142] Pino IP Nightingale TE Hoover C Zhao Z Cahalan M Dorey TW . The safety of epidural spinal cord stimulation to restore function after spinal cord injury: post-surgical complications and incidence of cardiovascular events. Spinal Cord. (2022) 60:903–10. doi: 10.1038/s41393-022-00822-w, 35701485

[143] Darrow DP Balser DY Freeman D Pelrine E Krassioukov A Phillips A . Effect of epidural spinal cord stimulation after chronic spinal cord injury on volitional movement and cardiovascular function: study protocol for the phase II open label controlled E-STAND trial. BMJ Open. (2022) 12:e059126. doi: 10.1136/bmjopen-2021-059126, 35851008 PMC9297213

[144] Courtine G Bloch J. Defining ecological strategies in Neuroprosthetics. Neuron. (2015) 86:29–33. doi: 10.1016/j.neuron.2015.02.039, 25856483

[145] Peters CG Harel NY Weir JP Wu Y-K Murray LM Chavez J . Transcutaneous spinal cord stimulation to stabilize seated systolic blood pressure in persons with chronic spinal cord injury: protocol development. Neurotrauma Rep. (2023) 4:838–47. doi: 10.1089/neur.2023.0063, 38156073 PMC10754346

[146] Shackleton C Samejima S Williams AM Malik RN Balthazaar SJ Alrashidi A . Motor and autonomic concomitant health improvements with neuromodulation and exercise (MACHINE) training: a randomised controlled trial in individuals with spinal cord injury. BMJ. (2023) 13:544. doi: 10.1136/bmjopen-2022-070544, 37451734 PMC10351300

[147] Hofstoetter US Freundl B Binder H Minassian K. Common neural structures activated by epidural and transcutaneous lumbar spinal cord stimulation: elicitation of posterior root-muscle reflexes. PLoS One. (2018) 13:e0192013. doi: 10.1371/journal.pone.0192013, 29381748 PMC5790266

[148] Phillips AA Squair JW Sayenko DG Edgerton VR Gerasimenko Y Krassioukov AV. An autonomic Neuroprosthesis: noninvasive electrical spinal cord stimulation restores autonomic cardiovascular function in individuals with spinal cord injury. J Neurotrauma. (2018) 35:446–51. doi: 10.1089/neu.2017.5082, 28967294 PMC5793952

[149] Engel-Haber E Bheemreddy A Bayram MB Ravi M Zhang F Su H . Neuromodulation in spinal cord injury using transcutaneous spinal stimulation—mapping for a blood pressure response: a case series. Neurotrauma Reports. (2024) 5:845–56. doi: 10.1089/neur.2024.0066, 39391052 PMC11462428

[150] Gerasimenko Y Gorodnichev R Moshonkina T Sayenko D Gad P Edgerton VR. Transcutaneous electrical spinal-cord stimulation in humans. Annals Physical Rehab Med. (2015) 58:225–31. doi: 10.1016/j.rehab.2015.05.003, 26205686 PMC5021439

[151] Solinsky R Burns K Tuthill C Hamner JW Taylor JA. Transcutaneous spinal cord stimulation and its impact on cardiovascular autonomic regulation after spinal cord injury. Am J Physiol Heart Circ Physiol. (2024) 326:H116–22. doi: 10.1152/ajpheart.00588.2023, 37947438 PMC11213470

[152] Mukhametova E Militskova A Biktimirov A Kharin N Semenova E Sachenkov O . Consecutive transcutaneous and epidural spinal cord neuromodulation to modify clinical complete paralysis—the proof of concept. Mayo Clinic Proceed Innovations, Quality Outcomes. (2024) 8:1–16. doi: 10.1016/j.mayocpiqo.2023.09.006, 38186923 PMC10770429

[153] West T Driver CN D'Souza RS. Incidence of Neuraxial and non-Neuraxial hematoma complications from spinal cord stimulator surgery: systematic review and proportional Meta-analysis. Neuromodulation. (2023) 26:1328–38. doi: 10.1016/j.neurom.2022.07.005, 35985940

[154] Khan H Kumar V Ghulam-Jelani Z McCallum SE Hobson E Sukul V . Safety of spinal cord stimulation in patients who routinely use anticoagulants. Pain Med. (2018) 19:1807–12. doi: 10.1093/pm/pnx305, 29186582 PMC6127233

[155] Cragg JJ Stone JA Krassioukov AV. Management of cardiovascular disease risk factors in individuals with chronic spinal cord injury: an evidence-based review. J Neurotrauma. (2012) 29:1999–2012. doi: 10.1089/neu.2012.2313, 22738320

[156] Fossey MPM Balthazaar SJT Squair JW Williams AM Poormasjedi-Meibod MS Nightingale TE . Spinal cord injury impairs cardiac function due to impaired bulbospinal sympathetic control. Nat Commun. (2022) 13:1382. doi: 10.1038/s41467-022-29066-1, 35296681 PMC8927412

[157] Hall ED Springer JE. Neuroprotection and acute spinal cord injury: a reappraisal. NeuroRx. (2004) 1:80–100. doi: 10.1602/neurorx.1.1.80, 15717009 PMC534914

[158] Kwon BK Okon E Hillyer J Mann C Baptiste D Weaver LC . A systematic review of non-invasive pharmacologic neuroprotective treatments for acute spinal cord injury. J Neurotrauma. (2011) 28:1545–88. doi: 10.1089/neu.2009.1149, 20146558 PMC3143410

